# Deficiency of miR-29a/b1 leads to premature aging and dopaminergic neuroprotection in mice

**DOI:** 10.3389/fnmol.2022.978191

**Published:** 2022-10-06

**Authors:** Xiaochen Bai, Jinghui Wang, Xiaoshuang Zhang, Yilin Tang, Yongtao He, Jiayin Zhao, Linlin Han, Rong Fang, Zhaolin Liu, Hongtian Dong, Qing Li, Jingyu Ge, Yuanyuan Ma, Mei Yu, Ruilin Sun, Jian Wang, Jian Fei, Fang Huang

**Affiliations:** ^1^Department of Translational Neuroscience, MOE Frontiers Center for Brain Science, Institutes of Brain Science, State Key Laboratory of Medical Neurobiology, Jing’an District Centre Hospital of Shanghai, Fudan University, Shanghai, China; ^2^Department of Neurology, Huashan Hospital, Fudan University, Shanghai, China; ^3^Department of Rehabilitation Medicine, Shanghai Jiao Tong University Affiliated Sixth People’s Hospital, Shanghai, China; ^4^Shanghai Engineering Research Center for Model Organisms, SMOC, Shanghai, China; ^5^School of Life Science and Technology, Tongji University, Shanghai, China

**Keywords:** Parkinson’s disease, miR-29a/b1, glial cells, neuroinflammation, AMPK

## Abstract

Parkinson’s disease (PD) is a neurodegenerative disorder characterized by progressive degeneration of midbrain dopaminergic neurons. The miR-29s family, including *miR-29a* and *miR-29b1* as well as *miR-29b2* and *miR-29c*, are implicated in aging, metabolism, neuronal survival, and neurological disorders. In this study, the roles of *miR-29a/b1* in aging and PD were investigated. *miR-29a/b1* knockout mice (named as 29a KO hereafter) and their wild-type (WT) controls were used to analyze aging-related phenotypes. After challenged with the neurotoxin 1-methyl-4-phenyl-1,2,3,6-tetrahydropyridine (MPTP), dopaminergic injuries, glial activation, and mouse behaviors were evaluated. Primary glial cells were further cultured to explore the underlying mechanisms. Additionally, the levels of miR-29s in the cerebrospinal fluid (CSF) of PD patients (*n* = 18) and healthy subjects (*n* = 17) were quantified. 29a KO mice showed dramatic weight loss, kyphosis, and along with increased and deepened wrinkles in skins, when compared with WT mice. Moreover, both abdominal and brown adipose tissues reduced in 29a KO mice, compared to their WT counterpart. However, in MPTP-induced PD mouse model, the deficiency of *miR-29a/b1* led to less severe damages of dopaminergic system and mitigated glial activation in the nigrostriatal pathway, and subsequently alleviated the motor impairments in 3-month-old mice. Eight-month-old mutant mice maintained such a resistance to MPTP intoxication. Mechanistically, the deficiency of *miR-29a/b-1* promoted the expression of neurotrophic factors in 1-Methyl-4-phenylpyridinium (MPP^+^)-treated primary mixed glia and primary astrocytes. In lipopolysaccharide (LPS)-treated primary microglia, knockout of *miR-29a/b-1* inhibited the expression of inflammatory factors, and promoted the expression of anti-inflammatory factors and neurotrophic factors. Knockout of *miR-29a/b1* increased the activity of AMP-activated protein kinase (AMPK) and repressed NF-κB/p65 signaling in glial cells. Moreover, we found miR-29a level was increased in the CSF of patients with PD. Our results suggest that 29a KO mice display the peripheral premature senility. The combined effects of less activated glial cells might contribute to the mitigated inflammatory responses and elicit resistance to MPTP intoxication in *miR-29a/b1* KO mice.

## Introduction

Parkinson’s disease (PD), characterized by progressive degeneration of midbrain dopaminergic neurons (Kikuchi et al., 2017), ranks second among the most common neurodegenerative disorders (Kam et al., 2018; Surguchov, 2022). Clinical motor symptoms are triggered by progressive loss of dopaminergic neurons in the substantia nigra pars compacta (SNpc) and consequently malnourished projection in the striatum (Kam et al., 2018). Also, increasing evidence indicates the role of gliosis and inflammatory response mechanisms followed by dopamine neuronal loss in the pathogenesis of PD (Martinez and Peplow, 2017).

miRNAs, small non-coding RNA molecules with only about 21 nucleotides in length, emerged as ideal powerful candidates for genetic programing (Kosik, 2006). They exert complex effects on target gene expression post-transcriptionally by degrading mRNA or repressing translation through targeting 3′ untranslated regions (UTR) of mRNA (Selbach et al., 2008). A diversity of miRNAs is exclusively abundant in the nervous system, where they could contribute to neuronal apoptosis, axonal path finding, neural plasticity, and particularly the development of neurological diseases (Kosik, 2006; Hebert et al., 2008; Roshan et al., 2014). Accumulated studies have pointed miRNAs dysfunction, including miR-29 family, exists in the pathology of neurodegenerative diseases (Kosik, 2006; Hebert et al., 2008; Roshan et al., 2014; Papadopoulou et al., 2015).

miR-29 family (miR-29s) consists of four members (miR-29a, miR-29b1, miR-29b2, and miR-29c), of which miR-29b1 and miR-29b2 share identical mature sequence (Roshan et al., 2014; Papadopoulou et al., 2015). All the members have highly conserved mature sequences and identical seed sequences, and miR-29a/b1 and miR-29b2/c are encoded by two genomic clusters on different chromosomes (Roshan et al., 2014; Papadopoulou et al., 2015). miR-29s, highly expressed in the brain, are implicated in aging, metabolism, neuronal survival, and neurological disorders (Ugalde et al., 2011; Papadopoulou et al., 2015; Caravia et al., 2018). Down-regulation of miR-29a/b1 was reported in neurodegenerative disorders, like Alzheimer’s disease (Hebert et al., 2008) and Huntington’s disease (Roshan et al., 2014). Simultaneously, our previous study revealed that miR-29s in the blood serum of patients with PD were significantly downregulated (Bai et al., 2017), but the mechanisms of miR-29s perturbations on PD progression are not clear.

In this study, by using *miR-29a/b1* knockout (miR-29a KO) mice, changes in periphery were investigated. Further a subacute regimen of 1-methyl-4-phenyl-1,2,3,6-tetrahydropyridine (MPTP) was applied to generate PD model. Damages of the nigrostriatal pathway, mouse behaviors and the underlying mechanisms were comprehensively assessed. Additionally, miR-29a levels were evaluated in the cerebrospinal fluid (CSF) of PD patients. miR-29a KO mice showed obvious premature aging, implied by weight loss, fat decreasing, kyphosis, muscle weakness, gait disorder, and wrinkle increasing and deepening. However, deficiency of *miR-29a/b1* brought about mitigation of dopaminergic injury and glial activation, and consequently the alleviated behavioral impairments.

## Materials and methods

### Patients and clinical assessments

The sporadic PD patients refrained from taking any anti-Parkinsonian medications and fasted for at least 12 h before CSF samples were taken. Control subjects fasted for 12 h before CSF samples were taken. The CSF was collected by standardized lumbar puncture procedures. Shipment and storage were performed according to the protocols from Parkinson Progression Marker Initiative (PPMI). The CSF were aliquoted (200 μl/tube), flash frozen, and stored at –80°C. All participants provided written informed consent in accordance with the Declaration of Helsinki. This study was approved by the Human Studies Institutional Review Board, Huashan Hospital, Fudan University. All methods were performed in accordance with the relevant guidelines and regulations.

### Animals and drug treatments

*miR-29a/b1* knockout mice (Liao et al., 2019) and their wild-type (WT) littermates (Shanghai Research Center for Model Organisms, China) were housed in a room with constant temperature (20–22°C) and light/dark cycle (12 h), and had free access to food and water. All experimental procedures were performed with the permission of the Institutional Animal Care and Use Committee of Fudan University, Shanghai Medical College. All surgeries were conducted under general anesthesia, and all efforts were made to minimize adverse effects and the number of animals used.

Using a subacute dosing regimen of MPTP (20 mg/kg in normal saline, Sigma, USA), mice were intraperitoneally treated with MPTP-HCl (Sigma, USA) in 0.9% NaCl or normal saline (NS) as control for five consecutive days at 24 h intervals as described (Selvaraj et al., 2012).

### RNA extraction and quantitative real-time-PCR

Total RNA from the tissue was extracted using TRIzol reagent (TIANGEN, China) following the manufacturer’s protocol. Reverse transcription was performed using random primers and the primers used in the qPCR are listed in Table 1. Relative expression levels were calculated using the comparative ΔΔCt method with β-actin as the normalizing control.

**TABLE 1:** Primers for quantitative real-time-PCR (qPCR) analysis.

Name	Sequence (5′→3′)
Mouse actin F	CAGGATGCAGAAGGAGATTAC
Mouse actin R	AACGCAGCTCAGTAACAGTC
Mouse BDNF F	TCATACTTCGGTTGCATGAAGG
Mouse BDNF R	AGACCTCTCGAACCTGCCC
Mouse CD14 F	GGACTGATCTCAGCCCTCTG
Mouse CD14 R	GCTTCAGCCCAGTGAAAGAC
Mouse Clcf1 F	CTTCAATCCTCCTCGACTGG
Mouse Clcf1 R	TACGTCGGAGTTCAGCTGTG
Mouse COX2 F	GTTCATCCCTGACCCCCAAG
Mouse COX2 R	ACTCTGTTGTGCTCCCGAAG
Mouse GDNF F	GACGTCATGGATTTTATTCAAGCCACC
Mouse GDNF R	CTGGCCTACTTTGTCACTTGTTAGCCT
Mouse Gbp2 F	GGGGTCACTGTCTGACCACT
Mouse Gbp2 R	GGGAAACCTGGGATGAGATT
Mouse Ggta1 F	GTGAACAGCATGAGGGGTTT
Mouse Ggta1 R	GTTTTGTTGCCTCTGGGTGT
Mouse H2-D1 F	TCCGAGATTGTAAAGCGTGAAGA
Mouse H2-D1 R	ACAGGGCAGTGCAGGGATAG
Mouse H2-T23 F	GGACCGCGAATGACATAGC
Mouse H2-T23 R	GCACCTCAGGGTGACTTCAT
Mouse IGF-1 F	AGAGCCTGCGCAATGGAATAAAGT
Mouse IGF-1 R	TTGGTGGGCAGGGATAATGAGG
Mouse IL-1β F	GCAACTGTTCCTGAACTC
Mouse IL-1β R	CTCGGAGCCTGTAGTGCA
Mouse IL-6 F	CATAGCTACCTGGAGTACATGA
Mouse IL-6 R	CATTCATATTGTCAGTTCTTCG
Mouse IL-10 F	AGCCGGGAAGACAATAACTG
Mouse IL-10 R	GGAGTCGGTTAGCAGTATGTTG
Mouse iNOS F	CCCTTCCGAAGTTTCTGGCAGCAGC
Mouse iNOS R	GGCTGTCAGAGCCTCGTGGCTTTGG
Mouse p21 F	GTGGGTCTGACTCCAGCCC
Mouse p21 R	CCTTCTCGTGAGACGCTTAC
Mouse p19^Arf^ F	GCCGCACCGGAATCCT
Mouse p19^Arf^ R	TTGAGCAGAAGAGCTGCTACGT
Mouse Pai1 F	TCAGAGCAACAAGTTCAACTACACTGAG
Mouse Pai1 R	CCCACTGTCAAGGCTCCATCACTTGCCCA
Mouse p53 F	GAGTATACCACCATCCACTACAAG
Mouse p53 R	GCACAAACACGAACCTCAAAG
Mouse S100α10 F	CCTCTGGCTGTGGACAAAAT
Mouse S100α10 R	CTGCTCACAAGAAGCAGTGG
Mouse Slc10α6 F	GCTTCGGTGGTATGATGCTT
Mouse Slc10α6 R	CCACAGGCTTTTCTGGTGAT
Mouse TGF-β1 F	CCTGAGTGGCTGTCTTTTGA
Mouse TGF-β1 R	CGTGGAGTTTGTTATCTTTGCTG
Mouse TNF-α F	CACGCTCTTCTGTCTACTGAACTTC
Mouse TNF-α R	GCAGCCTTGTCCCTTGAAGAGAACC
Mouse YM1 F	GTCACAGGTCTGGCAATTC
Mouse YM1 R	GTAGAGACCATGGCACTG

### miRNA extraction, reverse transcription, and quantitative polymerase chain reaction

Total RNA including miRNA from the CSF was extracted using miRNeasy Serum/Plasma Kit (Qiagen, Germany) following the manufacturer’s protocol. Then, 5 μl of total RNA was reverse transcribed using a miRcute miRNA First-Strand cDNA Synthesis Kit (Tiangen, China). Subsequently, 2 μl of the product was used to detect miR29s expression by quantitative real-time PCR (qPCR) using a miRcute miRNA qPCR Detection kit (Tiangen, China). The PCR primer sequences were as follows: miR-29a (5′-TAGCACCATCTGAAATCGG-3′); miR-29b (5′-TAGCACCATTTGAAATCAGT-3′); miR-29c (5′-TAGCACCATTTGAAATCGG-3′). Relative expression levels were calculated using the comparative ΔΔCt method with cel-miR-39 as the normalizing control.

### Protein extraction and western blot analysis

Mouse brain tissues or cell pellets were lysed in protein extraction reagents supplemented with a protease inhibitor cocktail. 30 μg of protein samples were separated by sodium dodecyl sulfate-polyacrylamide gels and then transferred onto polyvinylidene difluoride membranes (Millipore, USA) as described previously (Wang et al., 2020). The primary and secondary antibodies used were listed in Supplementary Table 1. The protein bands were detected with an Odyssey infrared imaging system (Li-Cor, USA). The relative expression levels of protein were quantified by densitometry analysis using Quantity One 4.5.2 software (Bio-Rad, Hercules, CA, USA). All the original images of Western blot were shown in the end of Supplementary Information section.

### Immunohistochemical staining and immunofluorescence staining

After anesthesia, mice were transcardially perfused with cold NS solution. Brains were harvested and post-fixed with 4% paraformaldehyde at 4°C overnight, and subsequently immersed in 20 and 30% sucrose solution at 4°C overnight. Embedded in the OCT compound, brains were cut into 30 μm thick coronal sections using a freezing microtome (Leica, Germany) and then stored in a cryoprotectant solution at 20°C. The primary and secondary antibodies used were listed in Supplementary Table 1.

For immunohistochemical staining, mouse brain sections were permeabilized, quenched the endogenous peroxidases with 0.3% H_2_O_2_ and blocked in phosphate-buffered saline with 0.2% Triton X-100 (PBS-T) containing 10% goat serum and then incubated at 37°C for 1 h. Then the sections were incubated with primary antibodies in PBS with 1% goat serum at 4°C overnight. After washing, the sections were incubated with biotinylated secondary antibodies at 37°C for 45 min and then with AB peroxidase (1:200; Vector Laboratories, USA) at 37°C for 45 min. The peroxidase reaction was detected with DAB Peroxidase (HRP) Substrate Kit (Vector Laboratories, USA).

For immunofluorescence staining, brain sections were blocked in PBS-T containing 10% goat serum and then incubated at 4°C overnight with the primary antibodies. After washing, the sections were incubated for 2 h at room temperature with secondary antibodies. All sections were counterstained with 4’,6-diamidino-2-phenylindole (DAPI) (400 ng/ml, Beyotime, China) for 5 min. Images were collected using an Olympus FV1000 confocal microscope (Japan).

### Stereological cell counting

The total numbers of tyrosine hydroxylase-positive (TH^+^) neurons in the SNpc were counted using the optical fractionator method on a Stereo Investigator system (Micro Brightfield, USA) attached to an (Olympus, Japan) as previously described (Liberatore et al., 1999). Briefly, one out of four 30 μm-thick sections and a total of six sections from bregma –2.80 to –3.65 mm were collected. The SN region was delineated using a 5× objective, and the actual counting was performed under a 40× objective. Stereological counting was performed in a double-blind fashion by two operators.

### Quantification of Iba1-, glial fibrillary acidic protein-positive cells

To measure the number of ionized calcium binding adapter molecule 1-positive (Iba1^+^) cells, and glial fibrillary acidic protein-positive (GFAP^+^) cells, we performed cell counting according to the published method and analyzed with Image-Pro Plus 6.0 (Media Cybernetics, USA) (Baiguera et al., 2012). Briefly, in both the SN and the dorsal striatum, two square 300 μm × 300 μm frames were placed and cell somata within the confines of the frames were counted. The positive cell numbers in the frames in each brain section divided by the volume of the region produced the cell density. Measurements from six sections were averaged to obtain one value per mouse. The observer blinded to experimental groups performed the analysis.

### Densitometric analysis

Densitometric analysis of TH-positive fibers in the striatum was performed as previously described (Huang et al., 2018). An average of six sections from bregma +1.60 to 0.00 mm were examined at 5× magnification with a light microscope (Leica, Germany). To determine the density of TH-immunoreactive staining in the striatum, a 700 μm × 700 μm frame was placed in the dorsal part of the striatum. Another 200 μm × 200 μm frame was placed in the corpus callosum to measure background values. The average of the background density readings from the corpus callosum was subtracted from the average of the density readings in the striatum for each section. Then, the average of all sections from each animal was calculated.

### High performance liquid chromatography

The striatum was dissected from the brain tissue, then weighed and sonicated in 0.4 M HClO_4_ on ice. The homogenate was centrifuged at 15,294 g at 4°C for 15 min. The supernatant was removed for determining the concentration of monoamines and their metabolite using the chromatograph (ESA, Chelmsford, MA, USA) with a 5014B electrochemical detector.

### Behavioral tests

#### Rotarod test

One day before the test, mice were given a training session the same as the test mode (4–40 rpm constant accelerating mode for 5 min) on the rotarod (MED Associates, USA) three times separated by 1 h intervals. All animals learned to perform. On the testing day, the time on the rod, with a maximum recording time of 300 s, was recorded according to the references (Hinkle et al., 2012; Liu et al., 2015). Data were collected from three trials separated by 1 h intervals. Then, the average time on the rod of all trials from each animal was calculated.

#### Wire hanging test

A 50 cm wide 2-mm thick metallic wire is secured to two vertical stands and the wire is maintained 35 cm above a layer of bedding material. One day before the test, mice were pre-trained three times separated by 30 min intervals. On the testing day, mice suspended by their forelimbs from the wire and subjected to a 180 s lasting hanging test. The suspended mice tended to support themselves with their hind paws to avoid falling and to walk along the wire to reach the platform. The number of falls (up to a maximum of 10) and reaches (up to a maximum of 10) during a period of 180 s were recorded. The test was carried out three times with 30 min intervals. An aggregate score from the number of falls and reaches was derived using the formula: (10-falls + reaches) (van Putten et al., 2011).

#### Grid hanging test

Mice were placed on a grid where it stood using all four limbs. Subsequently, the grid was turned upside down 35 cm above the home cage filled with bedding. One day before the test, mice were given a training session three times at 10 min intervals. All mice learned to perform. On the testing day, the trial ended after a hanging time of 3 min was achieved. The latency to when the animal falls is recorded. The test was carried out three times with 10 min intervals, the final results were an average of the three trials as previously described (van Putten et al., 2012).

#### Gait measurement

One day before the test, mice received a training session consisted of three trials to habituate to the CatWalk XT gait analysis system (Noldus, Netherlands). Rest between trials was approximately 1 h. Each animal was allowed an uninterrupted crossing of the recording field of the runway (length of approximately 40 cm) in both directions with three independent attempts at a 60% variation threshold. A high-speed camera carried out data acquisition and the software automatically classified the paw prints. Overall, runs for analysis were selected based on a minimum of five step cycles in the crossing field (Chen and Wu, 2022). After classification of the footprints in the CatWalk software, data were exported for external analysis using the Prism 7 software (GraphPad Software Inc., San Diego, CA, USA).

#### Rearing test

To assess the motor behavior, mice were placed individually in 400 ml glass beaker and the number of rearing events were recorded for 3 min. The beaker was cleaned with 75% ethanol between each animal.

### Primary astrocyte and microglia cell cultures

Primary glial cells were prepared from miR-29s deficient mice and their WT littermates at P1–P3, as described previously (Shao et al., 2013). The brains were dissected and meninges were removed in D-Hanks’ solution rapidly. Then brains were thoroughly snipped and trypsinized (0.25% trypsin) at 37°C for 5 min followed by termination with dulbecco’s modified eagle medium (DMEM) medium containing 10% fetal bovine serum (FBS). The cells were plated in 75 cm^2^ flask in DMEM medium with 10% FBS. Culture media were changed 48 h later to complete medium and subsequently twice a week. After 2 weeks, astrocytes were separated from microglia by shaking at 200 rpm for 12 h. Before experimental treatments, astrocytic cultures were plated in a six-well-plate at a density of 1 × 10^6^/well. The preliminary culture of microglia cells was the same as that of primary astrocytes, but the purification of microglia cells were prepared as described previously (Saura et al., 2003). Briefly, at day 21 *in vitro*, cultures were mildly trypsinized (0.0625% trypsin in D-Hanks’ solution) at 37°C for 30–40 min. Floating cells (mainly astrocytes) were removed. Microglia cells were cultured with supernatant of mixed glia cells. Before experimental treatments, microglia were plated in a twenty-four well-plate at a density of 3 × 10^5^/well.

### Multiplex immunoassay

ProcartaPlex kit (Thermo Fisher, USA) was used to measure the concentrations of cytokines/chemokines in the culture supernatants of primary astrocytes and microglia and homogenates of mouse striatum according to the manufacturer’s instructions. Briefly, 50 μl cell culture supernatants or 25 μl tissue homogenates were added into each filter plate well containing conjugated antibody beads, then the plate was incubated at room temperature for 30 min at 500 rpm and 4°C overnight. After washing, the beads were incubated with detection antibody and subsequently SA-PE at 500 rpm for 30 min, respectively, at room temperature. The beads were re-suspended in reading buffer and then the signals were detected by Luminex 200 (Luminex, USA).

### X-ray microCT scans

A whole-body micro-computed tomography (microCT) scan was performed to visualize adipose tissue and skeleton of mice. The detailed three-dimensional images of the structure of mice were obtained by high resolution X-ray microCT scanning (Quantum FX; PerkinElmer, USA) (Liu et al., 2018). Mice were anesthetized with 0.8% pentobarbital sodium (the volume was 10 times of their body weight), and put on the platform. The current and voltage were set to 74 μA and 70 kVp. It took about 4 min per mouse. Image segmentation was conducted using a volume-editing tool, and volumes were quantified using the region of interest module within the software package (AnalyzeDirect, USA).

### Statistical analysis

Data are presented as the means ± SEM. Statistical analyses were performed using Prism 7 software (GraphPad Software Inc., USA). Statistical significance was determined using two-tailed unpaired Student’s *T*-test for comparisons between two groups or Two-way analysis of variance (ANOVA) followed by least significant difference (LSD) for comparisons among more groups. *P* < 0.05 was considered statistically significant.

## Results

### Accelerated aging in the periphery of *miR-29a/b1* KO mice

**FIGURE 1:**
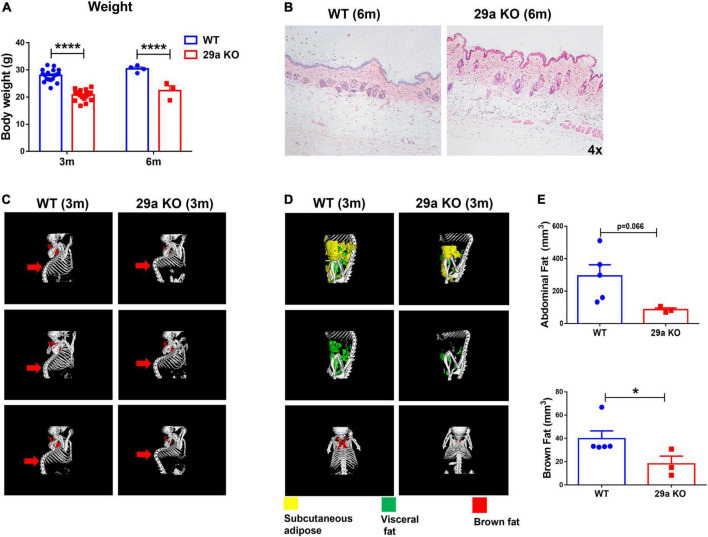
Peripheral characteristics of *miR-29a/b1* KO mice. **(A)** The body weights of wild-type (WT) and 29a KO mice at 3 and 6 months old. *n* = 3–18. Differences were analyzed by Student-*T*-test. ****p < 0.0001. **(B)** H&E staining of the back skin of WT and 29a KO mice at 6 months old. **(C)** microCT scan of bone of WT and 29a KO mice at 3 months old. **(D)** microCT scan of abdominal fat (subcutaneous fat and visceral fat together) and brown fat of WT and 29a KO mice at 3 months old. **(E)** The content of abdominal and brown fat of WT and 29a KO mice at 3 months old. *n* = 3–5 are shown. Differences were analyzed by Student-*T*-test. *p < 0.05.

*miR-29a/b1* knockout mice (29a KO) were constructed by the method of CRISPR-Cas9 (Liao et al., 2019). The strategy and the results of genotyping of mutant mice were shown in Supplementary Figure 1. At 3 and 6 months old, the body weights of 29a KO mice were reduced significantly compared to their wild type counterpart (Figure 1A). Six-month-old 29a KO mice developed apparent dermis thickening, along with increased and deepened wrinkles shown by hematoxylin and eosin (H&E) staining (Figure 1B). Mouse bone and fat tissues were analyzed by X-Ray micro-computed tomography (microCT) scan. At 3 months old, 29a KO mice displayed obvious kyphosis (Figure 1C). Abdominal fat (subcutaneous fat and visceral fat together) and brown fat decreased in 3-month-old 29a KO mice compared to their WT littermate (Figures 1D,E). In brain, the transcripts of aging marker *p21*, but not *p53*, increased in the hippocampus of 29a KO mice at 6 months old. The expression levels of *p21* and *p53* did not alter in the cortex of 29a KO and WT mice. In addition, p53 and p16 proteins in the hippocampus of 29a KO mice showed no difference compared to their WT controls (Supplementary Figure 2).

### Muscle weakness and abnormal walking in *miR-29a/b1* KO mice

**FIGURE 2:**
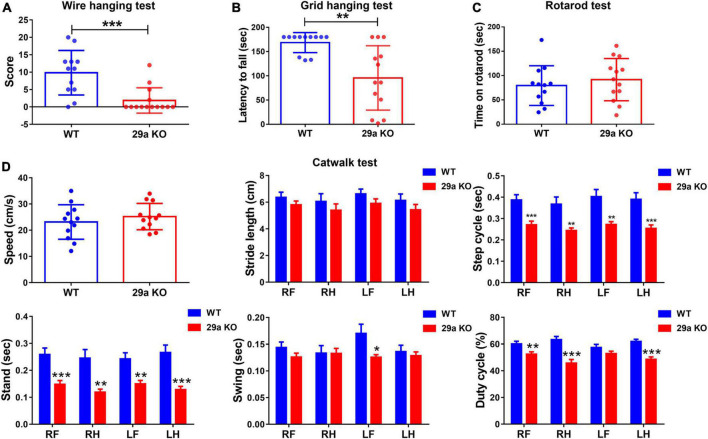
Muscle weakness and abnormal walking in *miR-29a/b1* KO mice. **(A)** The results of Wire hanging test of wild-type (WT) and 29a KO mice. *n* = 12–14. **(B)** The results of Grid hanging test in WT and 29a KO mice. *n* = 12–13. **(C)** The results of Rotarod test in WT and 29a KO mice. *n* = 12–13. **(D)** The results of Catwalk test in WT and 29a KO mice. *n* = 12; Differences were analyzed by Student-*T*-test. *P < 0.05, **P < 0.01, and ***P < 0.001.

Next, we evaluated whether deficiency of miR-29a/b1 led to behavioral changes. Wire hanging test and Grid hanging test were performed to measure the muscle strength. 29a KO mice gained lower scores in the Wire hanging test, indicated reduced forelimb strength (Figure 2A). In Grid hanging test, mutant mice showed shorter latency before falling compared to their WT counterparts (Figure 2B). However, there was no difference between WT and 29a KO mice in the Rotarod test (Figure 2C). Mouse gait was assessed by Catwalk XT gait analysis system. The speed and stride length of 29a KO and WT mice were close, however, the step cycle, stand and swing time were shorter and the duty cycle was significantly decreased, in mutant mice (Figure 2D).

### 1-Methyl-4-phenyl-1,2,3,6-tetrahydropyridine-induced damages of the nigrostriatal pathway are alleviated in mice with *miR-29a/b1* deficiency 3 days post-injection

**FIGURE 3:**
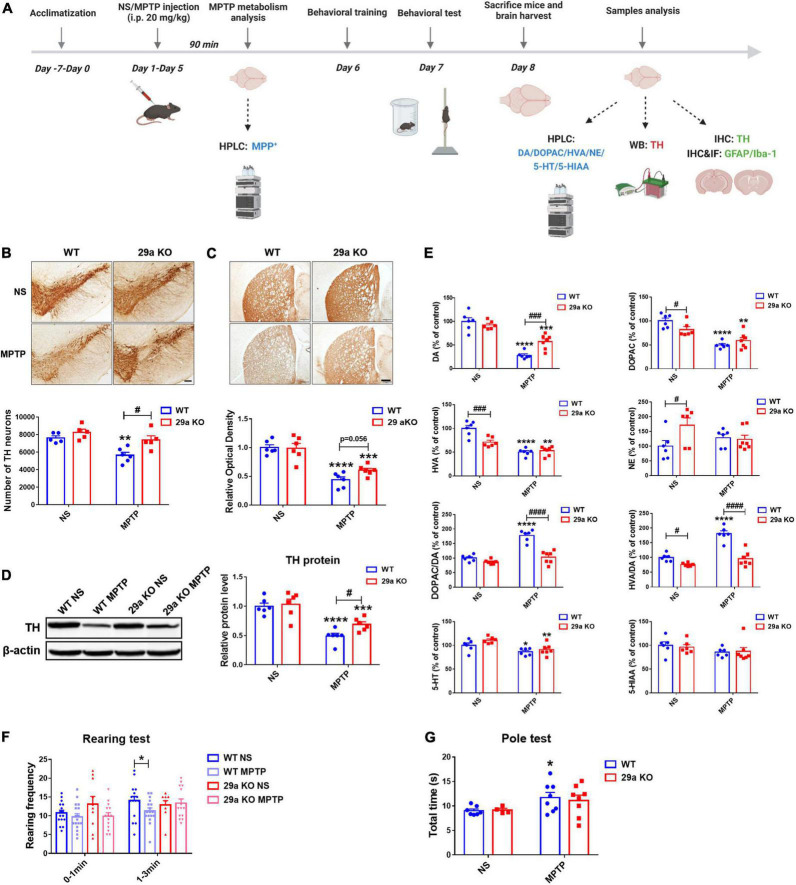
Analysis of the nigrostriatal pathway and behavioral performance of wild-type (WT) and *miR-29a/b1* KO mice after 1-methyl-4-phenyl-1,2,3,6-tetrahydropyridine (MPTP) administration. **(A)** The experimental schedule diagram created with BioRender.com. **(B)** Immunohistochemical staining of tyrosine hydroxylase (TH) in the substantia nigra pars compacta (SNpc) of WT and miR-29a KO mice at 3 days after MPTP administration. Scale bar: 0.1 mm. Stereological counting of TH positive dopaminergic neurons is shown in the lower panel. *n* = 5–6. **(C)** Immunohistochemical staining showing striatal TH positive nerve fibers of WT and 29a KO mice at 3 days after MPTP administration. Scale bar: 0.02 mm. Densitometric analysis of the relative optical density of the staining is shown in the lower panel. *n* = 6. **(D)** Western blot showing TH protein levels in the striatum of WT and miR-29a KO mice at 3 days after MPTP administration. β-actin served as a loading control. The quantification of the relative TH protein levels is shown in the right panel. *n* = 6. **(E)** Levels of striatal dopamine, 5-HT, their metabolites and norepinephrine (NE) in WT and miR-29a KO mice at 3 days after MPTP administration. *n* = 6–7. **(F)** The rearing frequency of WT and 29a KO mice between 0–1 and 1–3 min in the Rearing test at 2 days after MPTP administration. *n* = 9–17. **(G)** The total time of WT and 29a KO mice in the Pole test. *n* = 4–8. Differences were analyzed by two-way ANOVA followed by LSD multiple comparison tests. *p < 0.05, **p < 0.01, ***p < 0.001, and ****p < 0.0001, *vs.* normal saline (NS) control. ^#^*p* < 0.05, ^###^*p* < 0.001, and ^####^*p* < 0.0001, *vs.* WT group.

A close association of miR-29s and PD has been revealed in our previous studies (Bai et al., 2017, 2021; Han et al., 2020). To address whether deficiency of miR-29a/b1 affected the progression of PD *in vivo*. Three-month-old *miR-29a/b1* knockout mice and their WT littermates received five consecutive intraperitoneal injections of MPTP or NS at 24 h intervals as shown in the experimental schedule diagram (Figure 3A). Deficiency of miR-29a/b1 had no effect on the metabolic rate of MPTP indicated by the concentration of 1-Methyl-4-phenylpyridinium (MPP^+)^ in the striatum 90 min after MPTP exposure (Supplementary Figure 3). 1-Methyl-4-phenyl-1,2,3,6-tetrahydropyridine did not alter the striatal expression of aging marker genes *p21*, *p53*, and *Pai 1* in the two genotypes of mice (Supplementary Figure 4). In MPTP-challenged mouse nigrostriatal pathway, TH^+^ dopaminergic neurons in the SNpc, TH^+^ nerve fiber density and TH protein levels in the striatum all decreased dramatically (Figures 3B–D), and consequently, striatal dopamine (DA) and its metabolite 3,4-dihydroxyphenylacetic acid (DOPAC) and homovanillic acid (HVA) were reduced (Figure 3E). However, MPTP-induced damages of the nigrostriatal pathways in 29a KO were markedly mitigated indicated by less severe loss of dopaminergic neurons in the SNpc and dopaminergic nerve terminals in the striatum, higher striatal TH protein levels and DA concentrations, and reduced changes in the ratios of DOPAC to DA and HVA to DA (Figure 3E). Notably, under physiological conditions, DOPAC itself and HVA to DA ratio were lower, NE level was higher in 29a KO mice compared to their WT counterpart. Moreover, 5-HT and its metabolite 5-hydroxyindoleacetic acid (5-HIAA) did not differ between the two genotypes of mice (Figure 3E). To know if the expression of miR-29s in mouse brain responses to the challenge of a subacute regimen of MPTP, miR-29s levels in the striatum, ventral midbrain and hippocampus were detected by qPCR. We found none of the levels of miR-29a, miR-29b, or miR-29c changed (Supplementary Figure 5).

### 1-Methyl-4-phenyl-1,2,3,6-tetrahydropyridine-induced behavioral impairments are mitigated in mice with *miR-29a/b1* deficiency

The effects of miR-29a/b1 deficiency on MPTP-induced behavioral impairment were further investigated. Rearing behavior test, a measurement of spontaneous vertical activity (Willard et al., 2015; Chinta et al., 2018), was performed for 3 min at 48 h after the last MPTP injection. In the last 2 min, a relatively stable period, rearing frequency of WT mice but not 29a KO mice was reduced after MPTP exposure (Figure 3F). Likewise, in the Pole test, a classical locomotor activity detection method in PD model (Kam et al., 2018), total time was noticeably elevated in WT mice after MPTP exposure, while it did not alter in 29a KO mice compared to their NS controls (Figure 3G).

### 1-Methyl-4-phenyl-1,2,3,6-tetrahydropyridine-induced glial activation in the nigrostriatal pathway is alleviated in mice with *miR-29a/b1* deficiency

**FIGURE 4:**
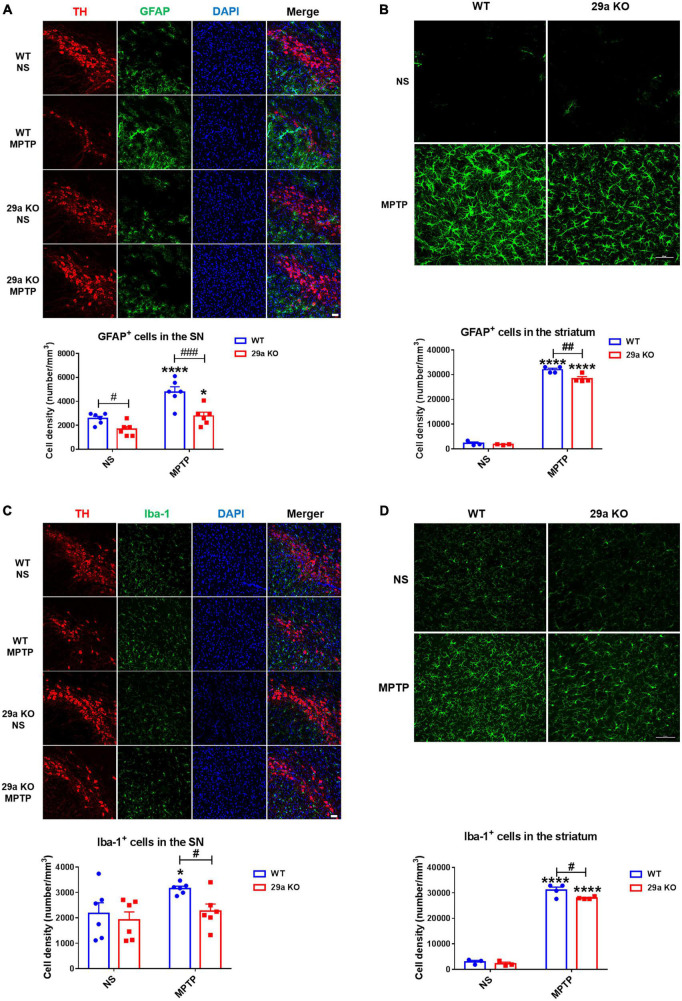
Analysis of glial activation in the nigrostriatal pathway at 3 days after 1-methyl-4-phenyl-1,2,3,6-tetrahydropyridine (MPTP) administration. **(A)** Immunofluorescence staining for tyrosine hydroxylase (TH) (red) and glial fibrillary acidic protein (GFAP) (green) in the substantia nigra pars compacta (SNpc) of wild-type (WT) and miR-29a KO mice. Scale bar: 0.1 mm. *n* = 6. **(B)** Immunofluorescence staining for GFAP (green) in the striatum of WT and miR-29a KO mice. Scale bar: 0.02 mm. *n* = 3–4. **(C)** Immunofluorescence staining for TH (red) and Iba-1 (green) in the SNpc of WT and 29a KO mice. Scale bar: 0.1 mm. Nuclei were counterstained with DAPI (Blue). *n* = 6. **(D)** Immunofluorescence staining for Iba-1 (green) in the striatum of WT and miR-29a KO mice. Scale bar: 0.05 mm. *n* = 3–4. Counting of GAFP^+^ cells and Iba-1^+^ cells in the striatum is shown in the lower panels. Difference was analyzed by two-way ANOVA followed by LSD multiple comparison tests. *p < 0.05 and ****p < 0.0001, *vs.* normal saline (NS) control. ^#^*p* < 0.05, ^##^*p* < 0.01, and ^###^*p* < 0.001, *vs.* WT group.

1-Methyl-4-phenyl-1,2,3,6-tetrahydropyridine induces glial cell activation in the nigrostriatal axis, and glial cells-mediated neuroinflammation exerts an important impact on PD pathology (Huang et al., 2017). At 3 days after MPTP administration, we assessed whether deficiency of *miR-29a/b1* influenced the activation of astrocytes and microglial cells in the SNpc and the striatum. Astrocytes increased dramatically in the SNpc and the striatum of both WT and 29a KO mice as revealed by immunofluorescence staining of GFAP and cell counting, however, astrocytic densities were significantly reduced in MPTP-treated 29a KO mice (Figures 4A,B). Likewise, Iba 1^+^ microglial cells increased in the SNpc and the striatum of WT mice, and in the striatum of 29a KO mice. Microglial densities were significantly decreased in the nigrostriatal axis of MPTP-treated 29a KO mice (Figures 4C,D). Moreover, pro-inflammatory cytokines interleukin-1β (IL-1β), IL-6, and interferon-γ (IFN-γ) were measured by multiplex immunoassay, they did not differ at baseline and 3 days after MPTP administration between the two genotypes of mice (Supplementary Figure 6).

### 1-Methyl-4-phenyl-1,2,3,6-tetrahydropyridine-induced damages in the nigrostriatal pathway is alleviated in older *miR-29a/b1* deficient mice

Deficiency of miR-29a/b1 led to pre-mature aging and dopaminergic protection. It was interesting to test the vulnerability of older mutant mice to MPTP-induced injury. Structurally, brains of 8-months-old 29a KO mice and their WT littermate were similar (Supplementary Figure 7). Three days after MPTP administration, the striatal TH protein levels in 29a KO mice were markedly higher compared to WT mice, whereas, GFAP proteins did not alter between the two genotypes of mice (Supplementary Figure 8).

### miR-29s expression responds to neurotoxin treatment in multiple types of cells

**FIGURE 5:**
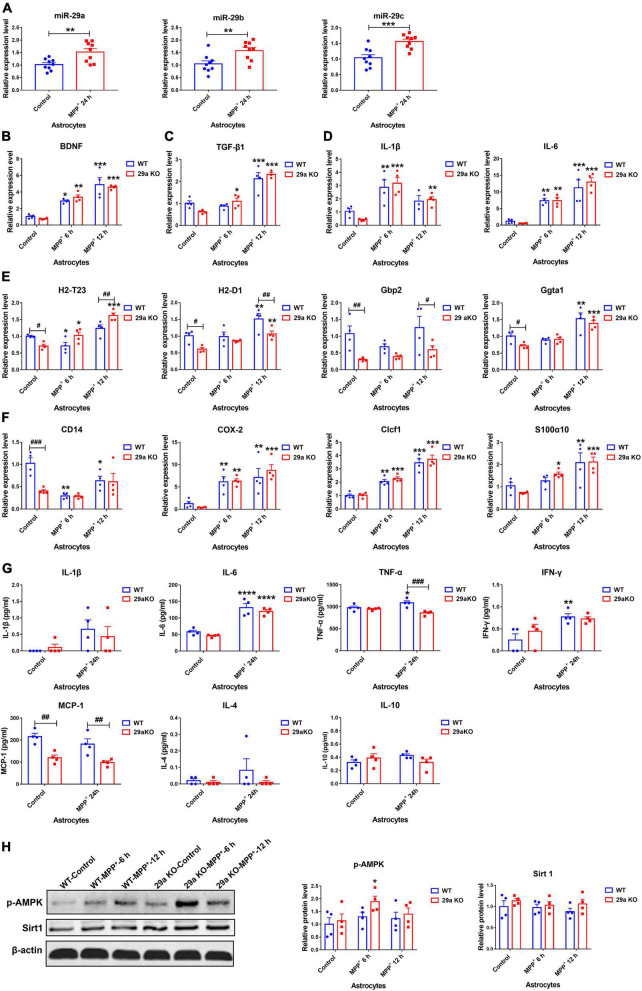
The expression levels of miR-29s and the effects of *miR-29a/b1* deficiency in 1-Methyl-4-phenylpyridinium (MPP^+)^-treated primary astrocytes. **(A)** The expression levels of miR-29s in primary astrocytes treated with phosphate-buffered saline (PBS) or 1 mM MPP^+^ for 24 h. *n* = 9. Differences were analyzed by Student-*T*-test. **p < 0.01 and ***p < 0.001. **(B–F)** qPCR analysis of neurotrophic factor *brain-derived neurotrophic factor (BDNF)* **(B)**, anti-inflammatory factor *TGF-*β*1* **(C)**, pro-inflammatory factors *IL-1*βand *IL-6* **(D)**, A1 marker genes *H2-T23, H2-D1, Gbp2*, and *Gpta1* **(E)**, A2 marker genes *CD14, COX-2, Clcf1*, and *S100*α*10* **(F)** transcripts in wild-type (WT) and 29a KO primary astrocytes treated with PBS or 1 mM MPP^+^ for 6 h and 12 h. *n* = 4. **(G)** The levels of inflammation-related cytokines IL-1β, IL-6, tumor necrosis factor-α (TNF-α), IFN-γ, MCP-1, IL-4, and IL-10 in the supernatants of WT and 29a KO primary astrocytes treated with PBS or 1 mM MPP^+^ for 24 h. *n* = 4. **(H)** Western blot analysis of p-AMPK and Sirt1 protein expression in WT and 29a KO primary astrocytes treated with PBS or 1 mM MPP^+^ for 6 h and 12 h. Quantifications of relative p-AMPK and sirt1 protein levels are shown in the right panel. *n* = 4. Differences were analyzed by two-way ANOVA followed by LSD multiple comparison tests. *p < 0.05, **p < 0.01, ***p < 0.001, and ****p < 0.0001, *vs.* PBS control. ^##^*p* < 0.01 and ^###^*p* < 0.001, *vs.* WT group.

Primary cultured microglial cells, astrocytes and midbrain neurons were challenged with 100 ng/ml LPS (for microglia) or 1 mM and 15 μM MPP^+^ (for astrocytes and neurons, respectively), miR-29s expression were then evaluated. We found the expression levels of miR-29s did not change in MPP^+^-treated midbrain neurons (Supplementary Figure 9A). However, all three members of miR-29s were upregulated in MPP^+^-treated primary astrocytes (Figure 5A). In LPS-treated primary microglia, their expression levels were downregulated, only miR-29a expression decreased significantly (Supplementary Figure 9B).

### Effects of *miR-29a/b1* deficiency in 1-methyl-4-phenylpyridinium-treated primary astrocytes

1-Methyl-4-phenylpyridinium exposure induced the expression of neurotrophic factors and inflammation-related genes in astrocytes. At 6, 12, and 24 h after the exposure, *brain-derived neurotrophic factor* (*BDNF*) transcripts increased in WT and 29a KO astrocytes. *Transforming growth factor-*β*1* (*TGF-*β*1*) transcript levels were dramatically elevated in 29a KO astrocytes after 6 h and 12 h treatment, and in WT astrocytes after 12 h treatment, and *insulin-like growth factor-1* (*IGF-1*) transcript only increased in 29a KO astrocytes after 24 h treatment (Figures 5B,C and Supplementary Figure 10A). Expression levels of *IL-1*β increased in WT astrocytes after 6 and 24 h treatment, and in 29a KO astrocytes after 6, 12, and 24 h treatment. *IL-6* transcripts were upregulated and did not differ in the astrocytes of two genotypes. *Inducible nitric oxide synthase* (*iNOS*) transcripts increased after 24 h treatment and did not vary between WT and 29a KO astrocytes (Figure 5D and Supplementary Figure 10B). *Tumor necrosis factor-*α (*TNF-*α) and *complement component 3* (*C3*) transcripts did not change after MPP^+^ treatment for 24 h (Supplementary Figure 10B). Activated astrocytes can be further divided into two subgroups: neurotoxic A1 type and neuroprotective A2 type. Here, we found A1 marker genes *H2-T23*, *H2-D1*, *Gbp2* and *Ggta1*, and A2 marker *CD14*, but not *cyclooxygenase-2* (*COX-2*), *Clcf1* and *S100*α*10*, were significantly lower in non-treated 29a KO astrocytes compared to WT control. At 6 h after the treatment, *H2-T23* and *CD14* decreased, *COX-2* and *Clcf1* increased, whereas *H2-D1*, *Gbp2*, *Ggta1*, and *S100*α*10* did not change in WT astrocytes; *H2-T23*, *COX-2*, *Clcf1*, and *S100*α*10* increased, whereas *H2-D1*, *Gbp2*, *Ggta1*, and *CD14* did not change in 29a KO astrocytes. At 12 h after the treatment, *CD14* decreased, *H2-D1*, *Gbp2*, *Ggta1*, *COX-2*, *Clcf1*, and *S100*α*10* increased, whereas *H2-T23* did not change in WT astrocytes; *H2-T23*, *H2-D1*, *Ggta1*, *COX-2*, *Clcf1*, and *S100*α*10* increased, whereas *Gbp2* and *CD14* had no alteration in 29a KO astrocytes. In addition, *H2-T23* transcripts were higher, whereas *H2-D1* and *Gbp2* transcripts were lower in 29a KO astrocytes compared to WT controls (Figures 5E,F). Pro-inflammatory cytokines including IL-1β, IL-6, TNF-α, IFN-γ, and monocyte chemoattractant protein-1 (MCP-1), anti-inflammatory cytokines IL-4 and IL-10 in the supernatants of primary astrocytes were measured at 24 h after MPP^+^ intoxication. Tumor necrosis factor-α and IFN-γ increased significantly in WT astrocytes, but not in 29a KO astrocytes after MPP^+^ treatment. Notably, TNF-α level in MPP^+^-treated 29a KO astrocytes was markedly lower compared to MPP^+^-treated WT astrocytes. Monocyte chemoattractant protein-1 levels in 29a KO astrocytes were downregulated compared to WT astrocytes at baseline and after MPP^+^ treatment. IL-6 levels were upregulated, whereas IL-1β, IL-4, and IL-10 did not change, in both WT and 29a KO astrocytes after the treatment of MPP^+^ (Figure 5G). By western blot assay, phosphorylated-AMPK protein level was increased in 29a KO astrocytes at 6 h after MPP^+^-treatment, while phosphorylated-AMPK protein level did not change in WT astrocytes after the treatment, and sirtuin 1 (Sirt1) protein levels did not alter between WT and 29a KO astrocytes (Figure 5H). Aging markers were further evaluated. *p19*, *p21*, *p16*, and *Pai1* transcript levels were increased in WT astrocytes at 24 h after MPP^+^ treatment, whereas only *p21* transcript, but not the other three increased in 29a KO astrocytes, and *p19* and *Pai1* transcript levels were even markedly lower in 29a KO astrocytes compared to WT controls (Supplementary Figure 11A). Moreover, anti-apoptotic Bcl-2 proteins did not alter in the two genotypes of primary astrocytes with or without MPP^+^ exposure (Supplementary Figure 11B).

### Effects of *miR-29a/b1* deficiency in lipopolysaccharide-treated primary microglial cells

**FIGURE 6:**
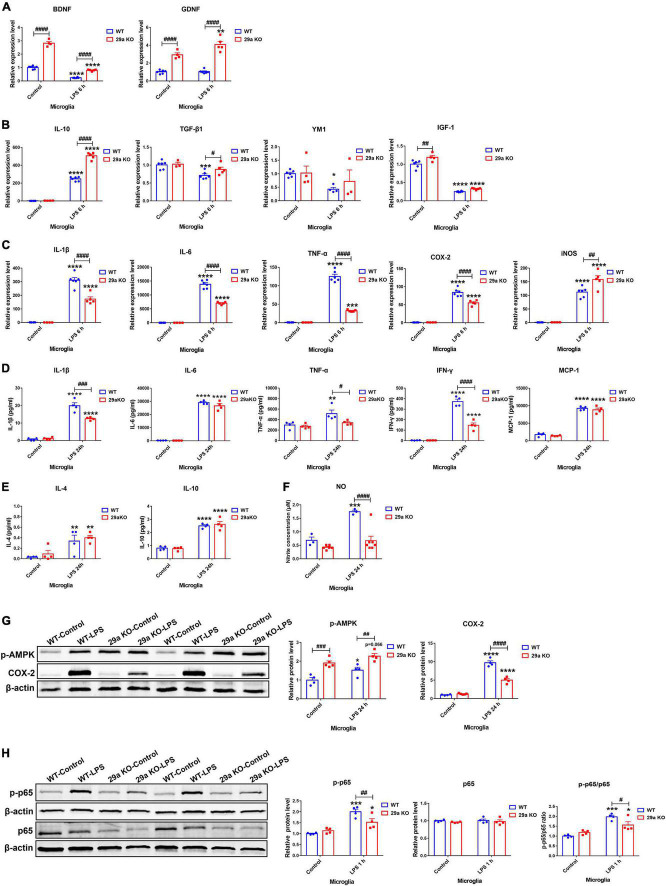
Effects of *miR-29a/b1* deficiency in LPS-treated primary microglia. **(A–C)** qPCR analysis of neurotrophic factors *brain-derived neurotrophic factor (BDNF)* and *GDNF* **(A)**, anti-inflammatory factors *IL-10, TGF-*β*1, YM1*, and insulin-like growth factor-1 (*IGF-1)* **(B)**, and pro-inflammatory factors *IL-1*β, *IL-6*, tumor necrosis factor-α (*TNF-*α), *COX2*, and inducible nitric oxide synthase (*iNOS)* **(C)** in wild-type (WT) and 29a KO primary microglia treated with phosphate-buffered saline (PBS) or 100 ng/ml LPS for 6 h. *n* = 3–6. **(D,E)** The levels of pro-inflammatory cytokines IL-1β, IL-6, TNF-α, IFN-γ, and MCP-1 **(D)**, and anti-inflammatory cytokines IL-4, and IL-10 **(E)**, in the supernatants of WT and 29a KO primary microglia treated with PBS or LPS for 24 h. *n* = 4. **(F)** The nitrite concentration in the supernatants of WT and 29a KO microglia treated with PBS or 100 ng/ml LPS for 24 h. *n* = 3–7. **(G)** Western blot analysis of p-AMPK and COX-2 protein expression in WT and 29a KO primary microglia treated with PBS or 100 ng/ml LPS for 24 h. Quantifications of relative p-AMPK and COX-2 are shown in the right panel. *n* = 4. **(H)** Western blot analysis of p-p65 and p65 protein expression in WT and 29a KO primary microglia treated with PBS or 100 ng/ml LPS for 1 h. Quantifications of relative p-p65 and p65 protein levels and their ratio are shown in the right panel. *n* = 4. Differences were analyzed by two-way ANOVA followed by LSD multiple comparison tests. *p < 0.05, **p < 0.01, ***p < 0.001, and ****p < 0.0001, *vs.* PBS control. ^#^*p* < 0.05, ^##^*p* < 0.01, ^###^*p* < 0.001, and ^####^*p* < 0.0001, *vs.* WT group.

Inflammation-provoking molecule LPS is widely used as a stimulator for microglia. In non-treated microglia, *BDNF*, *glial cell line-derived neurotrophic factor* (*GDNF*) and *IGF-1* transcripts were markedly increased in 29a KO microglia compared to WT control (Figures 6A,B). At 6 h after LPS treatment, transcripts of pro-inflammation genes *IL-1*β, *IL-6*, *TNF-*α, *COX-2*, and *iNOS*, and anti-inflammation gene *IL-10* were increased, those of *BDNF* and *IGF-1* were decreased in both WT and 29a KO microglia, whereas, expression levels of anti-inflammation genes *YM1* and *TGF-*β*1* decreased, *GDNF* transcript did not change in WT microglia. Likewise, *GDNF* transcript increased, and *YM1* and *TGF-*β*1* did not alter in 29a KO microglia after LPS challenge. Moreover, the transcripts of *BDNF*, *GDNF*, *IL-10*, *TGF-*β*1*, *iNOS* were significantly higher, and *IL-1*β, *IL-6*, *TNF-*α, and *COX-2* was lower in LPS-treated 29a KO microglia compared to WT control (Figures 6A–C). Pro-inflammatory cytokines IL-1β, IL-6, IFN-γ and MCP-1, anti-inflammatory cytokine IL-4 and IL-10, were significantly upregulated in the supernatants of LPS-treated WT and 29a KO primary microglia, TNF-α level was elevated only in WT microglia after LPS treatment; however, levels IL-1β, TNF-α and IFN-γ were dramatically reduced in 29a KO microglia compared to WT controls at 24 h after the treatment of LPS (Figures 6D,E). In addition, nitrite product was elevated in WT microglia, but not in 29a KO microglia at 24 h after LPS treatment (Figure 6F). By western blot, phosphorylated-AMPK (p-AMPK) protein levels were markedly upregulated in 29a KO microglia compared to WT microglia at baseline and 24 h after LPS administration. COX-2 proteins were increased in two genotypes of microglia, however, COX-2 protein level in 29a KO microglia was obviously reduced compared to WT microglia, at 24 h after LPS intoxication (Figure 6G). At 60 min after LPS treatment, phosphorylated-p65 (p-p65) and the ratio of p-p65 to p65, but not p65, were elevated in both WT and 29a KO microglia, however, p-p65 and the ratio were significantly reduced in 29a KO microglia compared to WT controls (Figure 6H).

### Effects of *miR-29a/b1* deficiency in 1-methyl-4-phenylpyridinium-treated primary mixed glia

**FIGURE 7:**
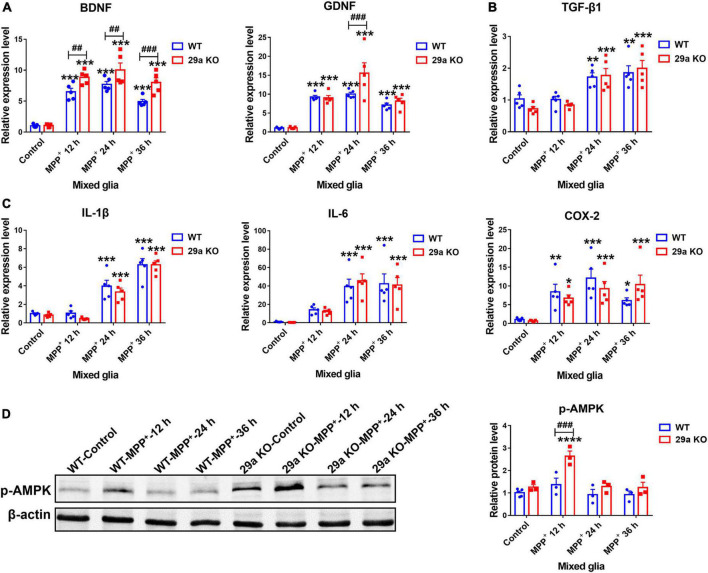
The effects of *miR-29a/b1* deficiency in 1-methyl-4-phenylpyridinium (MPP^+)^-treated primary mixed glia. qPCR analysis of neurotrophic factor *brain-derived neurotrophic factor (BDNF)*, *GDNF* **(A)**, anti-inflammatory factor *TGF-*β*1* **(B)** and pro-inflammatory factor *IL-1*β, *IL-6*, and *COX-2* **(C)** transcripts in wild-type (WT) and 29a KO primary mixed glia treated with phosphate-buffered saline (PBS) or 1 mM MPP^+^ for 12, 24, and 36 h. *n* = 5. **(D)** Western blot analysis of p-AMPK protein expression in WT and 29a KO primary mixed glia treated with PBS or 1 mM MPP^+^ for 12, 24, and 36 h. Quantification of relative p-AMPK is shown in the right panel. *n* = 3–4. Differences were analyzed by two-way ANOVA followed by LSD multiple comparison tests. *p < 0.05, **p < 0.01, ***p < 0.001, and ****p < 0.0001, *vs.* PBS control. ^##^*p* < 0.01 and ^###^*p* < 0.001, *vs.* WT group.

1-Methyl-4-phenylpyridinium treatment increased the expression of neurotrophic factor *BDNF*, *GDNF*, anti-inflammatory factor *TGF-*β*1*, and pro-inflammatory *IL-1*β, *IL-6*, and *COX-2* as well, in both WT and 29a KO primary mixed glia. At 12, 24, and 36 h after the treatment, the increases of *BDNF* transcripts were more dramatic in 29a KO mixed glia, also was the increase of *GDNF* at 24 h, compared to WT mixed glia. The transcripts of *TGF-*β*1*, *IL-1*β, *IL-6*, and *COX-2* did not differ between the primary mixed glia of the two genotypes (Figures 7A–C). By Western blot assay, we found phosphorylated-AMPK protein level in 29a KO mixed glia was upregulated after a 12 h-treatment of MPP^+^ compared to PBS control and MPP^+^-treated WT mixed glia (Figure 7D).

### Expression of miR-29s in the cerebrospinal fluid of Parkinson’s disease patients and healthy subjects

**FIGURE 8:**
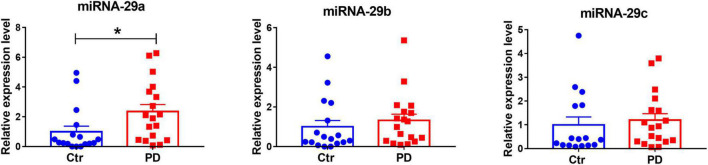
The expression levels of miR-29s in the cerebrospinal fluid (CSF) of control subjects and patients with Parkinson’s disease (PD). miR-29s levels in the CSF of control subjects and PD patients. *n* = 17–18. Differences were analyzed by Mann–Whitney test. *p < 0.05.

Our previous study has revealed decreasing miR-29s levels in blood serum of PD patients (Bai et al., 2017). Here through quantitative PCR, we measured miR-29s levels in the CSF of PD patients and healthy subjects. Demographic and clinical profiles of PD patients and control groups were in Table 2. We found that miR-29a, but not miR-29b and miR-29c, was upregulated in the CSF of PD patients (Figure 8). Moreover, there were no differences in the CSF levels of miR-29s between drug-naive PD patients and PD patients with medication (Data not shown).

**TABLE 2:** Demographic and clinical profiles of Parkinson’s disease (PD) patients and control groups.

	Controls	PD	Hoehn & Yahr stage I	Hoehn & Yahr stage II	Hoehn & Yahr stage III
No. of subjects	17	18	5	11	2
Age (years)	61.88 ± 6.314	61.22 ± 6.44	62.2 ± 4.207	60.09 ± 7.648	65
F/M	9/8	10/8	4/1	5/6	1/1
Disease duration (mo)		38.83 ± 42.02	34.4 ± 16.09	42.18 ± 51.64	31.5 ± 44.55
Levodopa equivalent dose (mg/day)		540 ± 323.5	300	587 ± 431.9	569 ± 8.49
No. of drug-naïve patients		11	4	7	0
MMSE		23.89 ± 5.04	21.4 ± 5.505	26.36 ± 2.501	16.5 ± 6.364

PD, Parkinson’s disease; mo, month; MMSE, Mini Mental State Examination. The data are presented as means ± SD.

## Discussion

*miR-29a/b1* gene locus is on chromosome six in mouse genome, nearby there is no protein-coding gene. In this study, roles of *miR-29a/b1* in aging and PD were investigated. We found that *miR-29 a/b1* null mutation leads to premature aging, however, mutant mice manifest dopaminergic neuroprotection after MPTP administration. Such characteristics are similar, but more pronounced compared to *miR-29 b2/c* knockout mice (Bai et al., 2021). miR-29 family members show increased expression in multiple tissues including brain, muscle, and liver during aging (Ugalde et al., 2011; Fenn et al., 2013; Hu et al., 2014). miR-29s upregulate p53 expression and induce cell cycle arrest (Varela et al., 2005; Park et al., 2009), and participate in p16/Rb-driven cellular senescence as well (Martinez et al., 2011). However, both pro-aging and anti-aging roles of miR-29s have been reported. miR-29s are induced during aging in short-lived turquoise killifish brain, where they elicit neuroprotection through inhibiting oxidative stress (Ripa et al., 2017). On the other hand, significant up-regulation of miR-29s contributes to aging-induced sarcopenia in rodents (Hu et al., 2014). Functions of miR29s in aging are very complex. Even within one single system, different functions of miR-29s have been explored (Boon et al., 2011; Heid et al., 2017).

Dooley et al. (2016) found 10 weeks old *miR-29a/b1^–/–^* mice exhibited reduced body weights and lengths, and had less white fat compared to the WT mice. Similarly, we observed that 8-week-old *miR-29a/b-1^–/–^* mice were shorter compared to their WT littermate (data not shown). 29a KO mice at 3 months old showed dramatic weight loss. Moreover, their abdominal fat (subcutaneous fat and visceral fat together) and brown fat all decreased. Aging-associated kyphosis was apparent in mutant mice. Changes in skin and muscle are markers of aging (Fong et al., 2004; Liu et al., 2019; Ahmed et al., 2020). Six-month-old 29a KO mice developed apparent thickening of dermis, along with increased and deepened wrinkles. Metalloproteinase Zmpste24-deficient mice at 16 weeks old completely loss the subcutaneous fat layer and develop muscle weakness (Pendas et al., 2002; Fong et al., 2004). Such lipodystrophy and muscle weakness were observed in *miR-29a/b1* knockout mice at 3 months old. However, in the brains of 29a KO mice, p53 and p16 protein levels did not alter compared to their WT littermate, and brains of WT and KO mice were structurally similar. Therefore, cellular senescence was not obvious in the brain of *miR-29a/b1* KO mice; Premature aging phenotypes of mutant mice mainly displayed in the periphery. Aging is defined as a multifactorial process that affects most of the biological functions of the organism and increases susceptibility to diseases and death (Ugalde et al., 2011). However, aging processes in different tissue/organs can be non-synchronized. Parkinson’s disease is thought as an age-related neurological disease. We found the premature phenotype and dopaminergic protection are not tied together in *miR-29a/b1* deficient mice.

Both beneficial and detrimental roles of miR-29 family have been reported in diseases of the central nervous system (CNS). The miR-29 family was significantly decreased in neuroblastoma and neuronal cells following oxygen and glucose deprivation/reperfusion (OGD/R) treatment (Cao et al., 2018; Wei et al., 2018), and miR-29a significantly increased in the resistant dentate gyrus, but decreased in the vulnerable CA1 region of the hippocampus after transient forebrain ischemia and short periods of reperfusion (Ouyang et al., 2013). Thus, miR-29s play a protective role in ischemic injury. Papadopoulou et al. (2015) have reported that *miR-29a/b1* knockout mice develop a progressive disorder characterized by locomotor impairment and ataxia. In this study, we found mutant mice exhibited posture instability. In the striatum of *miR-29a/b1* KO mice, the concentrations of HVA and DOPAC, but not DA, were reduced, and the ratio of HVA to DA also decreased, indicating a reduction of DA metabolism. Enzymes monoamine oxidase-B (MAO-B), MAO-A and catechol-O-methyltransferase (COMT) are responsible for the metabolism of DA. By Western blot, striatal MAO-B and MAO-A proteins did not differ between WT and 29a KO mice (data not shown). The reduction of DA metabolism might be due to the insufficiency of COMT proteins in mutant mice, and warrants further studies.

After challenged with MPTP, 29a KO mice showed lower vulnerability of the dopaminergic system, and behavioral resistance to some extent. Survival of dopaminergic neurons is regulated by glial cells. Astrocytes and microglia produce multiple neuron-supporting neurotrophic factors. Meanwhile, they are the major innate immune cells in the CNS, and involve in the progression of neuroinflammation and PD. miR-29s expression did not alter in MPP^+^-exposed midbrain neurons, however, MPP^+^ induced the expression of all three members of miR-29s in primary cultured astrocytes, and miR-29s expression, especially miR-29a, was downregulated in LPS-treated primary microglial cells. The results suggest that in different types of cells, the response of miR-29s to stimuli varies.

In MPP^+^-challenged mixed glia culture, deficiency of *miR-29a/b1* increased the expression of *BDNF* and *GDNF*. Moreover, MPP^+^ enhanced the expression of *IGF-1* in *miR-29a/b1* mutant astrocytes, while *Pai 1* transcript, a molecule involved in aging and pro-inflammation, was inhibited in MPP^+^-treated 29a KO astrocytes. The secretion of pro-inflammation cytokines TNF-α and MCP-1 was also repressed in 29a KO astrocytes after the treatment of MPP^+^. Additionally, MPP^+^ stimulation resulted in complex changes in A1 and A2 markers depended on the duration and particular markers *per se*. That reactive astrocytes are grouped into types of A1 and A2 is worthy of further studies. In primary microglial culture, deficiency of *miR-29a/b1* mitigated LPS-induced inflammatory response, and simultaneously promoted the transcriptional expression of anti-inflammation cytokines and neurotrophic factors. The secretion of pro-inflammation cytokines IL-1β, TNF-α, and IFN-γ reduced in LPS-treated 29a KO microglia compared to LPS-treated WT microglia. There are more than one thousand predicted target genes of miR-29s. miR-29s and many of the predicted target genes are involved in the cell survival and metabolism processes (Dooley et al., 2017; Massart et al., 2017; Caravia et al., 2018; Kwon et al., 2019). PI3K p85 and AKT3, targets of miR-29s are critical for cell survival. The protein levels of these molecules were not changed in the striatum of 29a KO mice (data not shown). As an essential metabolic regulator, adenosine monophosphate-activated protein kinase (AMPK) pathway was further investigated. In all three types of primary glial cultures, phosphorylated AMPK protein levels were upregulated in mutant cells. Under LPS treatment, phosphorylated NF-κB p65 subunit and the ratio of p-p65 to p65 were reduced in *miR-29a/b1* mutant microglial cells. Activation of AMPK can further stimulate Sirtuin 1, and indirectly inhibit NF-κB pathway and the expression of downstream inflammation-related target genes (Salminen et al., 2011). Adenosine monophosphate-activated protein kinase activation has been reported to be neuroprotective in MPTP- induced PD mice (Lu et al., 2016). Our experimental results suggested that knockout of *miR-29a/b1* gene increased the activity of AMPK, which might subsequently inhibit the activation of NF-κB pathway and the inflammatory responses in microglia, and might consequently inhibit the astrocyte activation. All in all, enhanced AMPK and/or reduced NF-κB p65 signaling might contribute to the milder inflammation response in *miR-29a/b1* mutant glial cells and the neuroprotection of nigrostriatal axis in *miR-29a/b1* deficient mice (Supplementary Figure 12).

miR-29s family is highly expressed in brain (Ugalde et al., 2011; Papadopoulou et al., 2015). We found that the expression of miR-29s in the ventral midbrain and striatum did not alter in MPTP-injected mice. In the CSF of patients with PD, miR-29a level was significantly elevated compared to healthy subjects, whereas in our previous studies, the serum levels of miR-29s were markedly downregulated in PD patients (Bai et al., 2017), and miR-29s were associated with cognitive impairment in PD as well (Han et al., 2020). Changes of miRNAs are not necessarily parallel among the brain tissues, the CSF and the serum of patients with neurodegenerative diseases. For examples, miR-29a is downregulated in the frontal cortex of AD patients (Shioya et al., 2010), it does not change in the serum of patients with AD in our previous study (Bai et al., 2017) and studies from other labs (Kiko et al., 2014). miRNAs are differentially expressed in CSF and serum of patients with PD (Burgos et al., 2014). miR-29a was upregulated in gyri cinguli of PD patients (Schulz et al., 2019), while its expression did not change in the frontal cortex of PD patients compared to healthy subjects (Shioya et al., 2010). The upregulation of miR-29a in the CSF is not specific to PD, miR-29a levels in the CSF of AD patients were significantly higher than in control subjects (Kiko et al., 2014; Müller et al., 2016). The differences of serum and CSF miR-29s levels might be attributed to the different origination, dysfunction of the blood-CSF barrier, dysregulation of miRNA transportation, and worthy of further studies.

There are some limitations of the study. The underlying mechanisms of *miR-29a/b1* deficiency causing resistance to MPTP intoxication in mice are broad and general. The biological significance of the elevated level of miR-29a in the CSF of PD patients is not much clear. Collectively, the present study shows that deficiency of *miR-29a/b1* leads to pre-mature aging in the periphery, however, such mutation maintains mouse brain in a low-inflammatory microenvironment, and elicits certain resistance to the dopaminergic neurotoxin in adult and older mice. The family of miR-29s undertakes quite close functions in mice.

## Data availability statement

The original contributions presented in the study are included in the article/Supplementary material, further inquiries can be directed to the corresponding author/s.

## Ethics statement

The studies involving human participants were reviewed and approved by Human Studies Institutional Review Board, Huashan Hospital, Fudan University. The patients/participants provided their written informed consent to participate in this study. The animal study was reviewed and approved by Institutional Animal Care and Use Committee of Fudan University, Shanghai Medical College.

## Author contributions

FH, JF, JW, and RS proposed and supervised the study. FH, JF, JW, RS, XB, JHW, and XZ designed the research studies, acquired the data, analyzed the data, and wrote the manuscript. YT and LH contributed to the sample collection and clinical characterization of the patients and analyzed data. XB, JHW, XZ, YH, JZ, RF, ZL, HD, QL, JG, MY, and YM conducted the experiments. All authors contributed to the interpretation of data, revision of the manuscript, and approved the submitted version.

## Abbreviations

29a KO: *miR-29a/b1* knockout mice
3 ′ UTR: 3 ′ untranslated regions
5-HIAA: 5-hydroxyindoleacetic acid
AMPK: adenosine monophosphate-activated protein kinase
BDNF: brain-derived neurotrophic factor
C3: complement component 3
CNS: the central nervous system
COMT: catechol-*O*-methyltransferase
COX-2: cyclooxygenase-2
CSF: cerebrospinal fluid
DOPAC: 3,4-dihydroxyphenylacetic acid
GDNF: glial cell line-derived neurotrophic factor
GFAP: glial fibrillary acidic protein
Iba-1: ionized calcium binding adapter molecule 1
IGF-1: insulin-like growth factor-1
IFN- γ: interferon- γ
IL-1 β: interleukin-1 β
IL-6: interleukin-6
iNOS: inducible nitric oxide synthase
HVA: homovanillic acid
LPS: lipopolysaccharide
MAO-A: monoamine oxidase-A
MAO-B: monoamine oxidase-B
MCP-1: monocyte chemoattractant protein-1
microCT: micro-computed tomography
MPP^+^: 1-methyl-4-phenylpyridinium
MPTP: 1-methyl-4-phenyl-1,2,3,6-tetrahydropyridine
miR-29s: miR-29 family
NS: normal saline
OGD/R: oxygen and glucose deprivation/reperfusion
p-AMPK: phosphorylated-AMP-activated protein kinase
PBS-T: phosphate-buffered saline with 0.2% Triton X-100
PD: Parkinson’s disease
PPMI: Parkinson Progression Marker Initiative
Sirt 1: sirtuin 1
SNpc: substantia nigra pars compacta
TGF- β 1: transforming growth factor- β 1
TH: tyrosine hydroxylase
TNF- α: tumor necrosis factor- α
WT: wild-type.

## References

[B1] Ahmed I. A. Mikail M. A. Zamakshshari N. Abdullah A. H. (2020). Natural anti-aging skincare: Role and potential. *Biogerontology* 21 293–310. 10.1007/s10522-020-09865-z 32162126

[B2] Bai X. Tang Y. Yu M. Wu L. Liu F. Ni J. (2017). Downregulation of blood serum microRNA 29 family in patients with Parkinson’s disease. *Sci. Rep.* 7:5411. 10.1038/s41598-017-03887-3 28710399 PMC5511199

[B3] Bai X. Zhang X. Fang R. Wang J. Ma Y. Liu Z. (2021). Deficiency of miR-29b2/c leads to accelerated aging and neuroprotection in MPTP-induced Parkinson’s disease mice. *Aging (Albany NY)* 13 22390–22411. 10.18632/aging.203545 34543233 PMC8507277

[B4] Baiguera C. Alghisi M. Pinna A. Bellucci A. De Luca M. A. Frau L. (2012). Late-onset Parkinsonism in NFkappaB/c-Rel-deficient mice. *Brain* 135(Pt 9) 2750–2765. 10.1093/brain/aws193 22915735 PMC3437025

[B5] Boon R. A. Seeger T. Heydt S. Fischer A. Hergenreider E. Horrevoets A. J. (2011). MicroRNA-29 in aortic dilation: Implications for aneurysm formation. *Circ. Res.* 109 1115–1119. 10.1161/circresaha.111.255737 21903938

[B6] Burgos K. Malenica I. Metpally R. Courtright A. Rakela B. Beach T. (2014). Profiles of extracellular miRNA in cerebrospinal fluid and serum from patients with Alzheimer’s and Parkinson’s diseases correlate with disease status and features of pathology. *PLoS One* 9:e94839. 10.1371/journal.pone.0094839 24797360 PMC4010405

[B7] Cao L. Zhang Y. Zhang S. Jiang T. P. Chen L. Liu J. (2018). MicroRNA-29b alleviates oxygen and glucose deprivation/reperfusion-induced injury via inhibition of the p53-dependent apoptosis pathway in N2a neuroblastoma cells. *Exp. Ther. Med.* 15 67–74. 10.3892/etm.2017.5410 29399057 PMC5766061

[B8] Caravia X. M. Fanjul V. Oliver E. Roiz-Valle D. Moran-Alvarez A. Desdin-Mico G. (2018). The microRNA-29/PGC1alpha regulatory axis is critical for metabolic control of cardiac function. *PLoS Biol.* 16:e2006247. 10.1371/journal.pbio.2006247 30346946 PMC6211751

[B9] Chen M. L. Wu R. M. (2022). Homozygous mutation of the LRRK2 ROC domain as a novel genetic model of parkinsonism. *J. Biomed. Sci.* 29 60. 10.1186/s12929-022-00844-9 35965315 PMC9375908

[B10] Chinta S. J. Woods G. Demaria M. Rane A. Zou Y. McQuade A. (2018). Cellular senescence is induced by the environmental neurotoxin paraquat and contributes to neuropathology linked to Parkinson’s disease. *Cell Rep.* 22 930–940. 10.1016/j.celrep.2017.12.092 29386135 PMC5806534

[B11] Dooley J. Garcia-Perez J. E. Sreenivasan J. Schlenner S. M. Vangoitsenhoven R. Papadopoulou A. S. (2016). The microRNA-29 family dictates the balance between homeostatic and pathological glucose handling in diabetes and obesity. *Diabetes* 65 53–61. 10.2337/db15-0770 26696639 PMC4876765

[B12] Dooley J. Lagou V. Garcia-Perez J. E. Himmelreich U. Liston A. (2017). miR-29a-deficiency does not modify the course of murine pancreatic acinar carcinoma. *Oncotarget* 8 26911–26917. 10.18632/oncotarget.15850 28460473 PMC5432306

[B13] Fenn A. M. Smith K. M. Lovett-Racke A. E. Guerau-de-Arellano M. Whitacre C. C. Godbout J. P. (2013). Increased micro-RNA 29b in the aged brain correlates with the reduction of insulin-like growth factor-1 and fractalkine ligand. *Neurobiol. Aging* 34 2748–2758. 10.1016/j.neurobiolaging.2013.06.007 23880139 PMC3779520

[B14] Fong L. G. Ng J. K. Meta M. Cote N. Yang S. H. Stewart C. L. (2004). Heterozygosity for Lmna deficiency eliminates the progeria-like phenotypes in Zmpste24-deficient mice. *Proc. Natl. Acad. Sci. U.S.A.* 101 18111–18116. 10.1073/pnas.0408558102 15608054 PMC536056

[B15] Han L. Tang Y. Bai X. Liang X. Fan Y. Shen Y. (2020). Association of the serum microRNA-29 family with cognitive impairment in Parkinson’s disease. *Aging (Albany NY)* 12 13518–13528. 10.18632/aging.103458 32649312 PMC7377865

[B16] Hebert S. S. Horre K. Nicolai L. Papadopoulou A. S. Mandemakers W. Silahtaroglu A. N. (2008). Loss of microRNA cluster miR-29a/b-1 in sporadic Alzheimer’s disease correlates with increased BACE1/beta-secretase expression. *Proc. Natl. Acad. Sci. U.S.A.* 105 6415–6420. 10.1073/pnas.0710263105 18434550 PMC2359789

[B17] Heid J. Cencioni C. Ripa R. Baumgart M. Atlante S. Milano G. (2017). Age-dependent increase of oxidative stress regulates microRNA-29 family preserving cardiac health. *Sci. Rep.* 7:16839. 10.1038/s41598-017-16829-w 29203887 PMC5715159

[B18] Hinkle K. M. Yue M. Behrouz B. Dachsel J. C. Lincoln S. J. Bowles E. E. (2012). LRRK2 knockout mice have an intact dopaminergic system but display alterations in exploratory and motor co-ordination behaviors. *Mol. Neurodegener.* 7:25. 10.1186/1750-1326-7-25 22647713 PMC3441373

[B19] Hu Z. Klein J. D. Mitch W. E. Zhang L. Martinez I. Wang X. H. (2014). MicroRNA-29 induces cellular senescence in aging muscle through multiple signaling pathways. *Aging (Albany NY)* 6 160–175. 10.18632/aging.100643 24659628 PMC4012934

[B20] Huang D. Wang Z. Tong J. Wang M. Wang J. Xu J. (2018). Long-term changes in the nigrostriatal pathway in the MPTP mouse model of Parkinson’s disease. *Neuroscience* 369 303–313. 10.1016/j.neuroscience.2017.11.041 29196026

[B21] Huang D. Xu J. Wang J. Tong J. Bai X. Li H. (2017). Dynamic changes in the nigrostriatal pathway in the MPTP mouse model of Parkinson’s disease. *Parkinson’s Dis.* 2017 1–7. 10.1155/2017/9349487 28831326 PMC5555011

[B22] Kam T. I. Mao X. Park H. Chou S. C. Karuppagounder S. S. Umanah G. E. (2018). Poly(ADP-ribose) drives pathologic alpha-synuclein neurodegeneration in Parkinson’s disease. *Science* 362:eaat8407. 10.1126/science.aat8407 30385548 PMC6431793

[B23] Kiko T. Nakagawa K. Tsuduki T. Furukawa K. Arai H. Miyazawa T. (2014). MicroRNAs in plasma and cerebrospinal fluid as potential markers for Alzheimer’s disease. *J. Alzheimers Dis.* 39 253–259. 10.3233/jad-130932 24157723

[B24] Kikuchi T. Morizane A. Doi D. Magotani H. Onoe H. Hayashi T. (2017). Human iPS cell-derived dopaminergic neurons function in a primate Parkinson’s disease model. *Nature* 548 592–596. 10.1038/nature23664 28858313

[B25] Kosik K. S. (2006). The neuronal microRNA system. *Nat. Rev. Neurosci.* 7 911–920. 10.1038/nrn2037 17115073

[B26] Kwon J. J. Factora T. D. Dey S. Kota J. (2019). A systematic review of miR-29 in cancer. *Mol. Ther. Oncolytics* 12 173–194. 10.1016/j.omto.2018.12.011 30788428 PMC6369137

[B27] Liao Y. Ouyang L. Ci L. Chen B. Lv D. Li Q. (2019). Pravastatin regulates host foreign-body reaction to polyetheretherketone implants via miR-29ab1-mediated SLIT3 upregulation. *Biomaterials* 203 12–22. 10.1016/j.biomaterials.2019.02.027 30851489

[B28] Liberatore G. T. Jackson-Lewis V. Vukosavic S. Mandir A. S. Vila M. McAuliffe W. G. (1999). Inducible nitric oxide synthase stimulates dopaminergic neurodegeneration in the MPTP model of Parkinson disease. *Nat. Med.* 5 1403–1409. 10.1038/70978 10581083

[B29] Liu J. Huang D. Xu J. Tong J. Wang Z. Huang L. (2015). Tiagabine protects dopaminergic neurons against neurotoxins by inhibiting microglial activation. *Sci. Rep.* 5:15720. 10.1038/srep15720 26499517 PMC4620555

[B30] Liu X. J. Duan N. N. Liu C. Niu C. Liu X. P. Wu J. (2018). Characterization of a murine nonalcoholic steatohepatitis model induced by high fat high calorie diet plus fructose and glucose in drinking water. *Lab. Invest.* 98 1184–1199. 10.1038/s41374-018-0074-z 29959418

[B31] Liu Y. Gao W. Koellmann C. Le Clerc S. Hüls A. Li B. (2019). Genome-wide scan identified genetic variants associated with skin aging in a Chinese female population. *J. Dermatol. Sci.* 96 42–49. 10.1016/j.jdermsci.2019.08.010 31522824

[B32] Lu M. Su C. Qiao C. Bian Y. Ding J. Hu G. (2016). Metformin prevents dopaminergic neuron death in MPTP/P-induced mouse model of Parkinson’s disease via autophagy and mitochondrial ROS clearance. *Int. J. Neuropsychopharmacol.* 19:yw047. 10.1093/ijnp/pyw047 27207919 PMC5043649

[B33] Martinez B. Peplow P. V. (2017). MicroRNAs in Parkinson’s disease and emerging therapeutic targets. *Neural Regen. Res.* 12 1945–1959. 10.4103/1673-5374.221147 29323027 PMC5784336

[B34] Martinez I. Cazalla D. Almstead L. L. Steitz J. A. DiMaio D. (2011). miR-29 and miR-30 regulate B-Myb expression during cellular senescence. *Proc. Natl. Acad. Sci. U.S.A.* 108 522–527. 10.1073/pnas.1017346108 21187425 PMC3021067

[B35] Massart J. Sjogren R. J. O. Lundell L. S. Mudry J. M. Franck N. O’Gorman D. J. (2017). Altered miR-29 expression in Type 2 diabetes influences glucose and lipid metabolism in skeletal muscle. *Diabetes* 66 1807–1818. 10.2337/db17-0141 28404597

[B36] Müller M. Jäkel L. Bruinsma I. B. Claassen J. A. Kuiperij H. B. Verbeek M. M. (2016). MicroRNA-29a is a candidate biomarker for Alzheimer’s disease in cell-free cerebrospinal fluid. *Mol. Neurobiol.* 53 2894–2899. 10.1007/s12035-015-9156-8 25895659 PMC4902829

[B37] Ouyang Y. B. Xu L. Lu Y. Sun X. Yue S. Xiong X. X. (2013). Astrocyte-enriched miR-29a targets PUMA and reduces neuronal vulnerability to forebrain ischemia. *Glia* 61 1784–1794. 10.1002/glia.22556 24038396 PMC3810393

[B38] Papadopoulou A. S. Serneels L. Achsel T. Mandemakers W. Callaerts-Vegh Z. Dooley J. (2015). Deficiency of the miR-29a/b-1 cluster leads to ataxic features and cerebellar alterations in mice. *Neurobiol. Dis.* 73 275–288. 10.1016/j.nbd.2014.10.006 25315682

[B39] Park S. Y. Lee J. H. Ha M. Nam J. W. Kim V. N. (2009). miR-29 miRNAs activate p53 by targeting p85 alpha and CDC42. *Nat. Struct. Mol. Biol.* 16 23–29. 10.1038/nsmb.1533 19079265

[B40] Pendas A. M. Zhou Z. Cadinanos J. Freije J. M. Wang J. Hultenby K. (2002). Defective prelamin A processing and muscular and adipocyte alterations in Zmpste24 metalloproteinase-deficient mice. *Nat. Genet.* 31 94–99. 10.1038/ng871 11923874

[B41] Ripa R. Dolfi L. Terrigno M. Pandolfini L. Savino A. Arcucci V. (2017). MicroRNA miR-29 controls a compensatory response to limit neuronal iron accumulation during adult life and aging. *BMC Biol.* 15:9. 10.1186/s12915-017-0354-x 28193224 PMC5304403

[B42] Roshan R. Shridhar S. Sarangdhar M. A. Banik A. Chawla M. Garg M. (2014). Brain-specific knockdown of miR-29 results in neuronal cell death and ataxia in mice. *RNA* 20 1287–1297. 10.1261/rna.044008.113 24958907 PMC4105753

[B43] Salminen A. Hyttinen J. M. Kaarniranta K. (2011). AMP-activated protein kinase inhibits NF-κB signaling and inflammation: Impact on healthspan and lifespan. *J. Mol. Med. (Berl.)* 89 667–676. 10.1007/s00109-011-0748-0 21431325 PMC3111671

[B44] Saura J. Tusell J. M. Serratosa J. (2003). High-yield isolation of murine microglia by mild trypsinization. *Glia* 44 183–189. 10.1002/glia.10274 14603460

[B45] Schulz J. Takousis P. Wohlers I. Itua I. O. G. Dobricic V. Rücker G. (2019). Meta-analyses identify differentially expressed micrornas in Parkinson’s disease. *Ann. Neurol.* 85 835–851. 10.1002/ana.25490 30990912

[B46] Selbach M. Schwanhausser B. Thierfelder N. Fang Z. Khanin R. Rajewsky N. (2008). Widespread changes in protein synthesis induced by microRNAs. *Nature* 455 58–63. 10.1038/nature07228 18668040

[B47] Selvaraj S. Sun Y. Watt J. A. Wang S. Lei S. Birnbaumer L. (2012). Neurotoxin-induced ER stress in mouse dopaminergic neurons involves downregulation of TRPC1 and inhibition of AKT/mTOR signaling. *J. Clin. Invest.* 122 1354–1367. 10.1172/jci61332 22446186 PMC3314472

[B48] Shao W. Zhang S. Z. Tang M. Zhang X. H. Zhou Z. Yin Y. Q. (2013). Suppression of neuroinflammation by astrocytic dopamine D2 receptors via alphaB-crystallin. *Nature* 494 90–94. 10.1038/nature11748 23242137

[B49] Shioya M. Obayashi S. Tabunoki H. Arima K. Saito Y. Ishida T. (2010). Aberrant microRNA expression in the brains of neurodegenerative diseases: miR-29a decreased in Alzheimer disease brains targets neurone navigator 3. *Neuropathol. Appl. Neurobiol.* 36 320–330. 10.1111/j.1365-2990.2010.01076.x 20202123

[B50] Surguchov A. (2022). “Biomarkers in Parkinson’s Disease,” in *Neurodegenerative diseases biomarkers: Towards translating research to clinical practice*, eds Peplow P. V. Martinez B. Gennarelli T. A. (New York, NY: Springer US), 155–180.

[B51] Ugalde A. P. Ramsay A. J. de la Rosa J. Varela I. Marino G. Cadinanos J. (2011). Aging and chronic DNA damage response activate a regulatory pathway involving miR-29 and p53. *EMBO J.* 30 2219–2232. 10.1038/emboj.2011.124 21522133 PMC3117645

[B52] van Putten M. Aartsma-Rus A. Louvain-Ia-Neuve L. (2011). *The use of hanging wire tests to monitor muscle strength and condition over time. Treat-Nmd.* Eu 1-12. Available online at http://www.treat-nmd.eu/downloads/file/sops/dmd/MDX/DMD_M.2.1.004.pdf (accessed October 8, 2016).

[B53] van Putten M. Kumar D. Hulsker M. Hoogaars W. M. Plomp J. J. van Opstal A. (2012). Comparison of skeletal muscle pathology and motor function of dystrophin and utrophin deficient mouse strains. *Neuromuscul. Disord.* 22 406–417. 10.1016/j.nmd.2011.10.011 22284942

[B54] Varela I. Cadinanos J. Pendas A. M. Gutierrez-Fernandez A. Folgueras A. R. Sanchez L. M. (2005). Accelerated ageing in mice deficient in Zmpste24 protease is linked to p53 signalling activation. *Nature* 437 564–568. 10.1038/nature04019 16079796

[B55] Wang Z. Dong H. Wang J. Huang Y. Zhang X. Tang Y. (2020). Pro-survival and anti-inflammatory roles of NF-κB c-Rel in the Parkinson’s disease models. *Redox Biol.* 30:101427. 10.1016/j.redox.2020.101427 31986466 PMC6994410

[B56] Wei R. Zhang R. Li H. Li H. Zhang S. Xie Y. (2018). MiR-29 targets PUMA to suppress oxygen and glucose deprivation/reperfusion (OGD/R)-induced cell death in hippocampal neurons. *Curr. Neurovasc. Res.* 15 47–54. 10.2174/1567202615666180403170902 29623838

[B57] Willard A. M. Bouchard R. S. Gittis A. H. (2015). Differential degradation of motor deficits during gradual dopamine depletion with 6-hydroxydopamine in mice. *Neuroscience* 301 254–267. 10.1016/j.neuroscience.2015.05.068 26067595 PMC4527082

