# Supplementary material for: Deficiency of miR-29a/b1 leads to premature aging and dopaminergic neuroprotection in mice
Source: Front Mol Neurosci. 2022 Oct 6;15:978191. doi: 10.3389/fnmol.2022.978191 (PMC9582353; doi:10.3389/fnmol.2022.978191)

Supplementary Information

Supplementary tables

Table S1. Information for primary and secondary antibodies

Name	Cat number	Supplier	Application
Rabbit anti-Bcl-2	12789-1-AP	Proteintech	WB:1:1000
Mouse anti β-actin	sc-70319	Santa Cruz	WB:1:1000
Rabbit anti-COX-2	Ab15191	Abcam	WB:1:1000
Mouse anti-NF-κB p65	sc-8008	Santa Cruz Biotechnology	WB:1:1000
Rabbit anti-phospho-NFκB p65	3033	Cell Signaling Technology	WB:1:1000
Rabbit anti-Sirt1 antibody	07-131	Millipore	WB:1:500
Rabbit phospho-AMPKα (Thr172)	2535	Cell Signaling Technology	WB:1:500
Rabbit anti-p16	211542	Abcam	WB:1:800
mouse anti -p53	2524	Cell Signaling Technology	WB:1:800
IRDye®R680LT Goat Anti-Mouse	92668070	LI-COR	WB:1:10000
IRDye®R800CW Goat Anti-Rabbit	92632211	LI-COR	WB:1:10000
Mouse anti-tyrosine hydroxylase	T2928	Sigma	IHC:1:1000 IF:1:500 WB:1:1000
Rabbit anti-GFAP	16825-1-AP	Proteintech	IHC:1:1000 IF:1:1000 WB:1:1000
Rabbit anti-Iba1	019-19741	Wako	IHC:1:500 IF:1:500
Goat anti-mouse Alexa Fluor 594	A11032	Invitrogen	IF:1:1000
Goat anti-rabbit Alexa Fluor 488	A32731	Invitrogen	IF:1:1000

Goat anti-mouse Alexa Fluor 647	A32728	Invitrogen	IF:1:1000
Biotinylated anti-mouse secondary antibody	PK-6102	Vector Laboratories	IHC:1:200
Biotinylated anti-rabbit secondary antibody	PK-6101	Vector Laboratories	IHC:1:200

Supplementary figures and legends

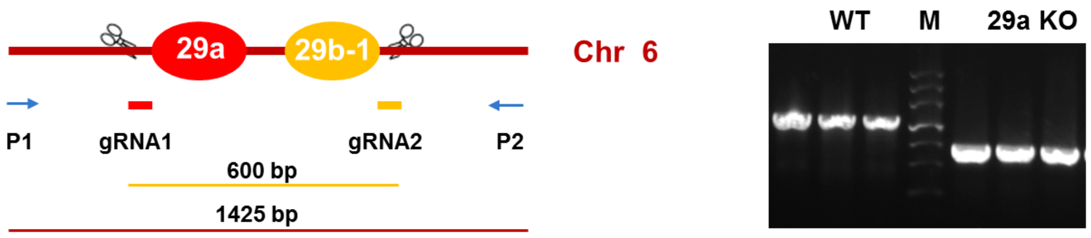

Figure S1. The strategy of *miR-29a/b1* knockout in mice and identification of 29a KO mice.

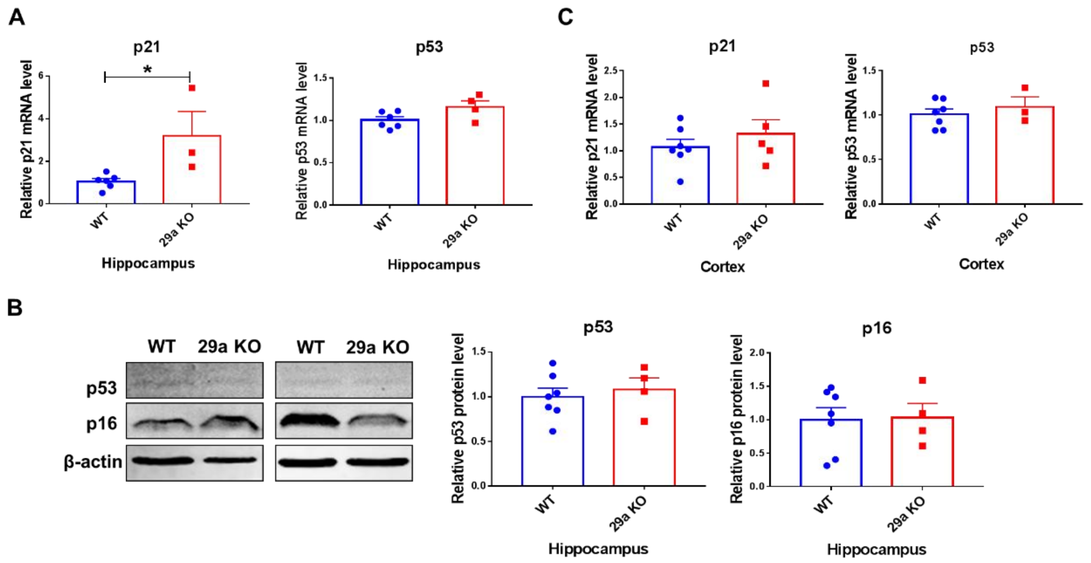

Figure S2. The expression of aging marker genes in the hippocampus and cortex of WT and miR-29a/b1 KO mice at 6 months old. (A) qPCR analysis of *p21* and *p53* transcripts in the hippocampus. Differences were analyzed by Student-T-test. n=3-5. *$p < 0.05$. **(B)** Western blot analysis of p53 and p16 protein expression in the hippocampus. Quantification of relative p53 and p16 expression levels are shown in the right panel. n=4-7. **(C)** qPCR analysis of *p21* and *p53* transcripts in the cortex. n=3-7.

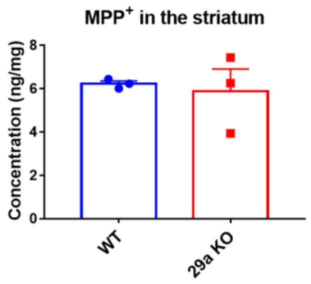

Figure S3. The concentration of MPP$^+$ in the striatum 90 min after the last MPTP injection. n=3.

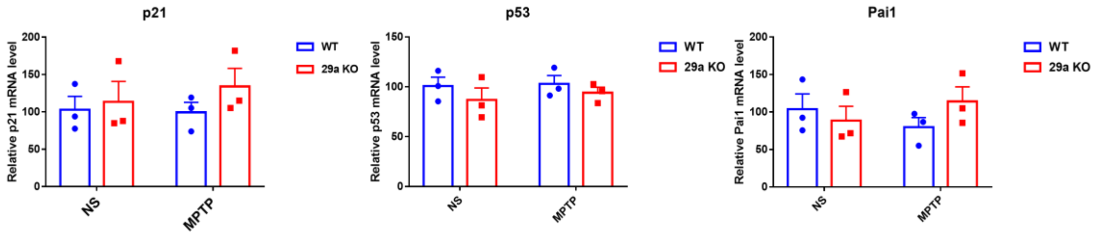

Figure S4. The expression levels of aging marker genes in the striatum of miR-29a/b1 KO mice. qPCR analysis of *p21*, *p53*, and *Pail-1* transcripts in the striatum of WT and 29a KO mice. n=3.

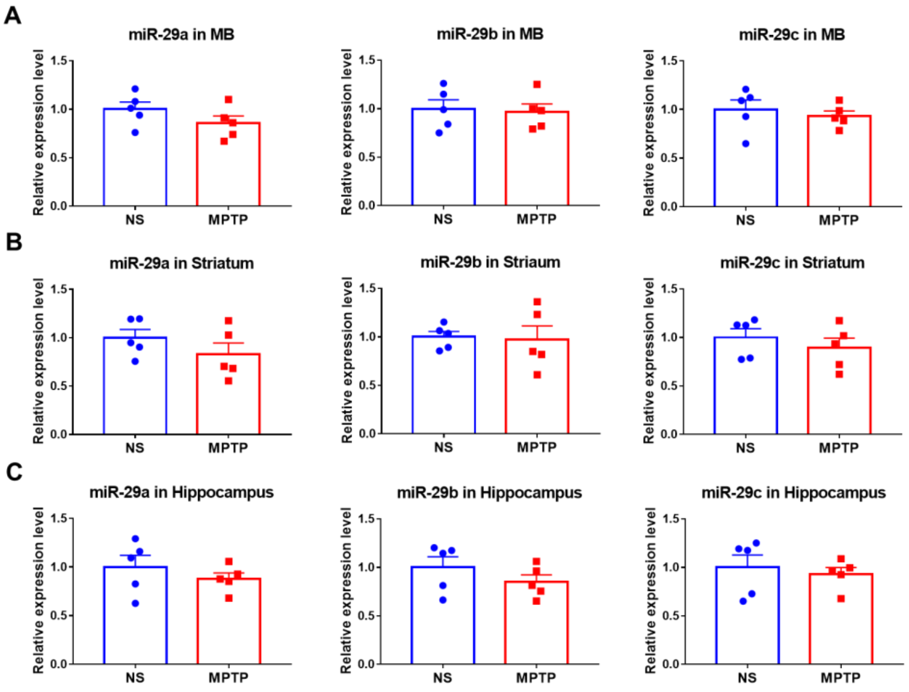

Figure S5. The expression levels of miR-29s in the ventral midbrain (MB) (A), striatum (B) and hippocampus (C) of mice at 3 days after MPTP administration. n=5.

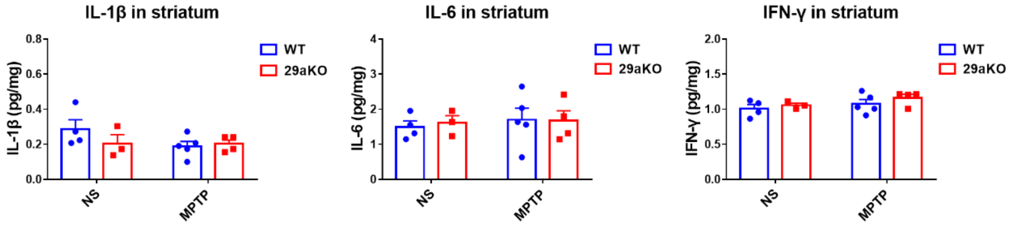

Figure S6. Cytokines IL-1β, IL-6 and IFN-γ in the striatum of WT and *miR-29a/b1* KO mice at 3 days after MPTP administration. n=3-5.

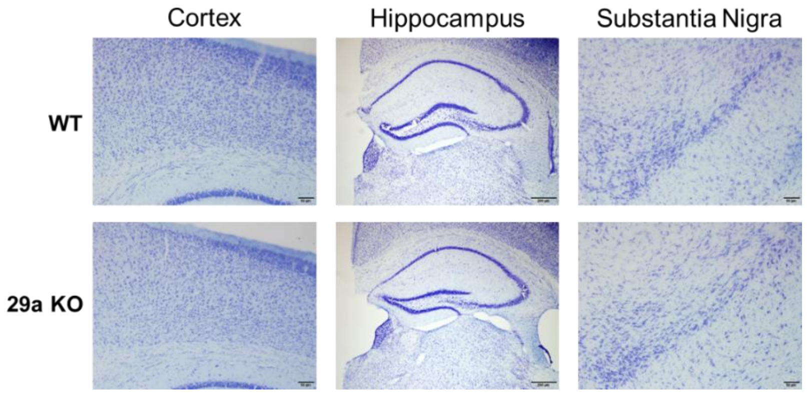

Figure S7. Nissl staining of the cortex, hippocampus and substantia nigra of 8-month-old WT and *miR-29a/b1* KO mice. Scale bar: 50 µm.

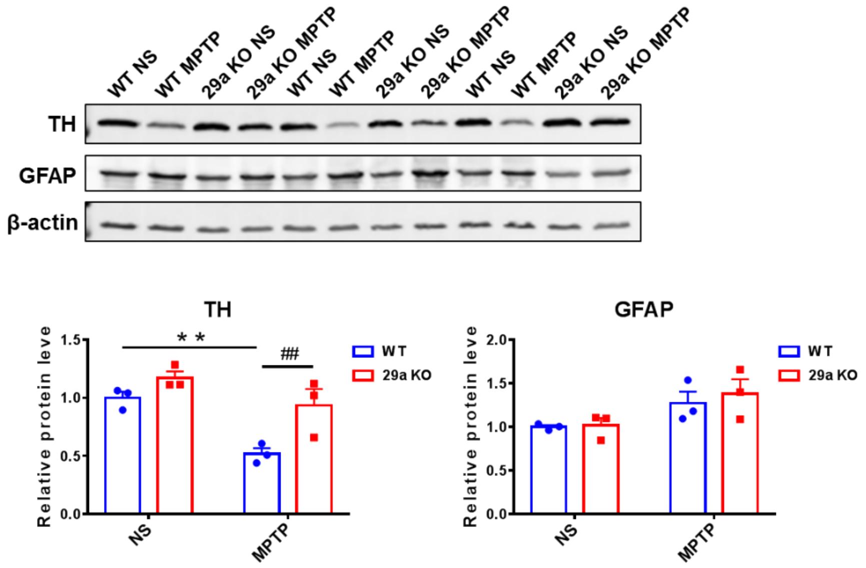

Figure S8. Western blot analysis of striatal TH and GFAP proteins in 8-month-old WT and *miR-29a/b1* KO mice at 3 days after a subacute regimen of MPTP intoxication. Quantification of relative TH and GFAP protein levels was shown at the bottom. n=3. Differences were analyzed by two-way ANOVA followed by LSD multiple comparison tests. **$p < 0.01$, *vs* normal saline control. ## $p<0.01$, *vs* WT group.

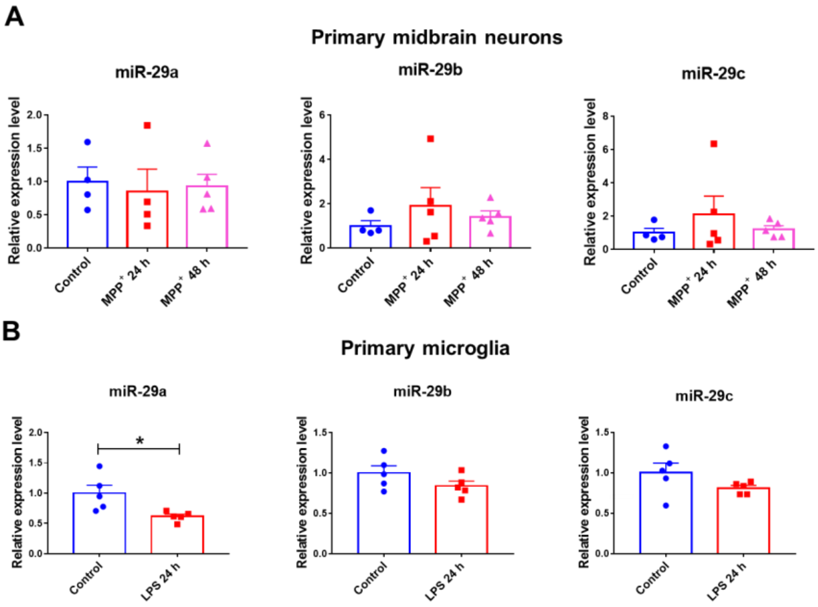

Figure S9. miR-29s levels in neurotoxin-treated primary midbrain neurons and microglial cells. (A) Expression of miR-29s in primary midbrain neurons treated with PBS or MPP^+ at 15 µM for 24 h and 48 h. n=4-5. **(B)** Expression of miR-29s in primary microglia treated with PBS or LPS at 100 ng/mL for 24 h. Scale bar: 100 µm. n=5. Differences were analyzed by Student-T-test. **p* < 0.05, ***p* < 0.01 and ****p* < 0.001.

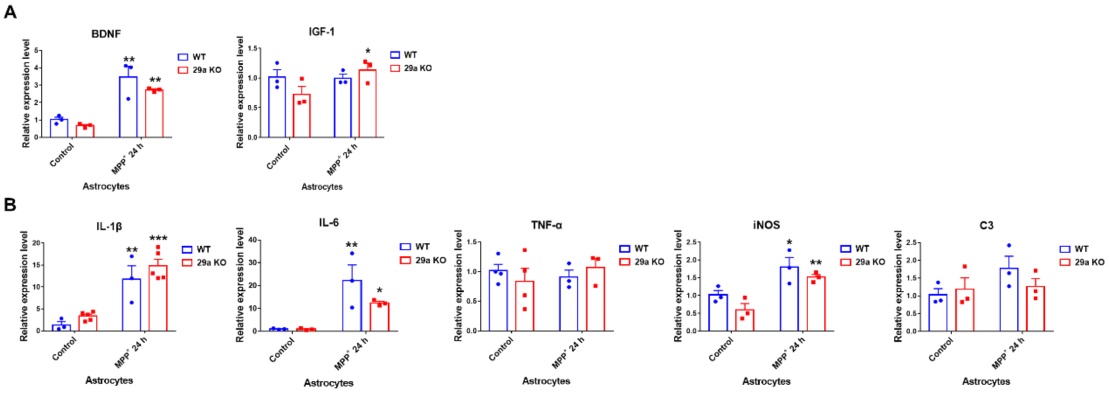

Figure S10. (A) qPCR analysis of *BDNF* and *IGF-1* transcripts and (B) *IL-1β*, *IL-6*, *TNF-α*, *iNOS* and *C3* in WT and miR-29a/b1 KO primary astrocytes treated with PBS or 1 mM MPP^+ for 24 h. n=3. **p*<0.05, ***p* < 0.01 and ****p*<0.001, *vs* PBS control.

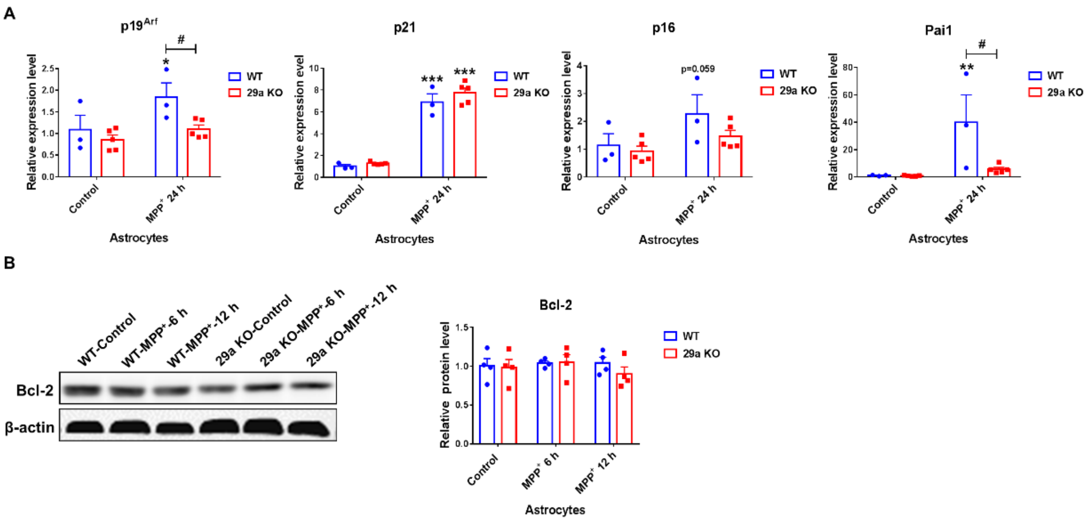

Figure S11. (A) qPCR analysis of aging markers *p19^{Arf}, p21, p16* and *Pai1* transcripts in WT and miR-29a/b1 KO primary astrocytes treated with PBS or 1 mM MPP$^+$ for 24 h. n=3-5. Differences were analyzed by two-way ANOVA followed by LSD multiple comparison tests. $*p < 0.05$, $**p < 0.01$ and $***p < 0.001$, *vs* PBS control. # $p < 0.05$, *vs* WT group. **(B) Western blot analysis of Bcl-2 protein expression in WT and 29a KO primary astrocytes treated with PBS or 1 mM MPP$^+$ for 6 h and 12 h.** Quantification of relative Bcl-2 protein level is shown in the right panel. n=4.

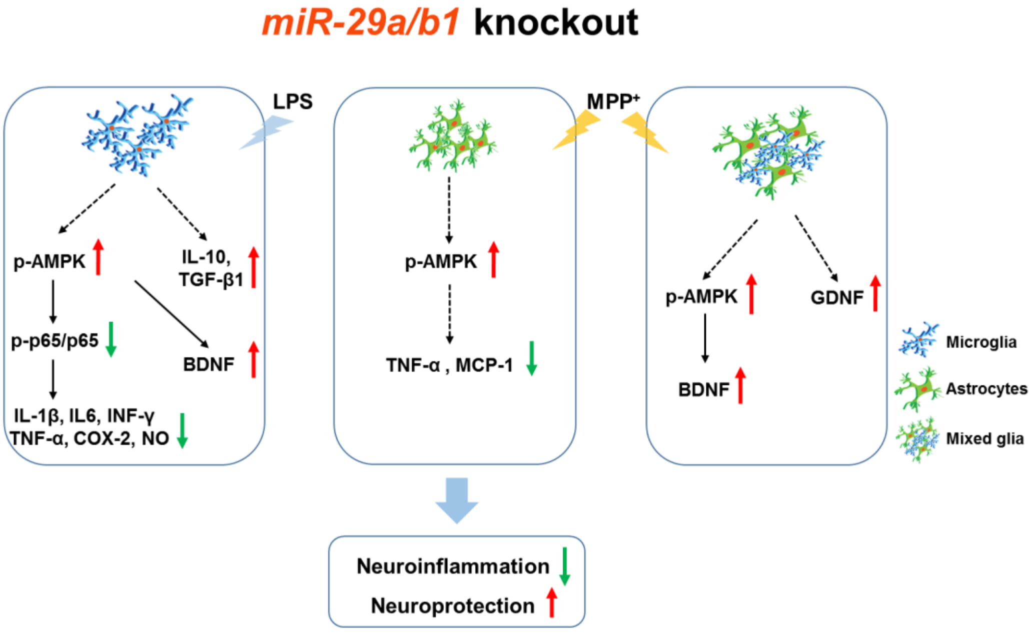

Figure S12. Diagram of effects of *miR-29a/b1* deficiency in Parkinson's disease.

The original membranes

1. **Original image for Figure 3C**

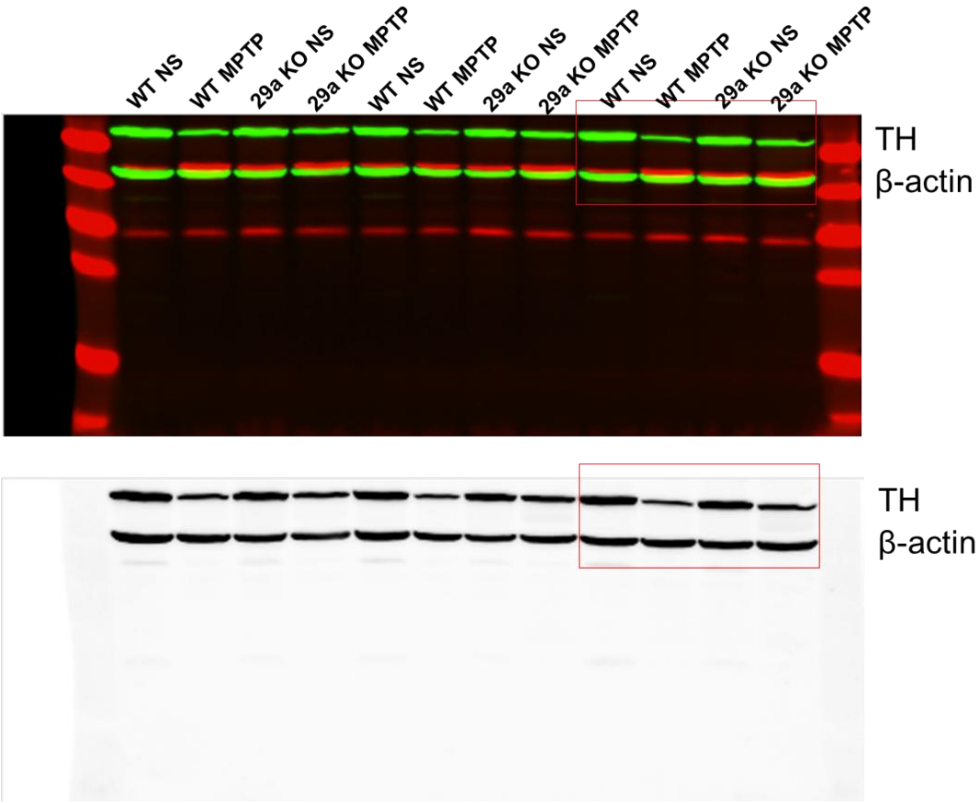

2. Original image for Figure 5H

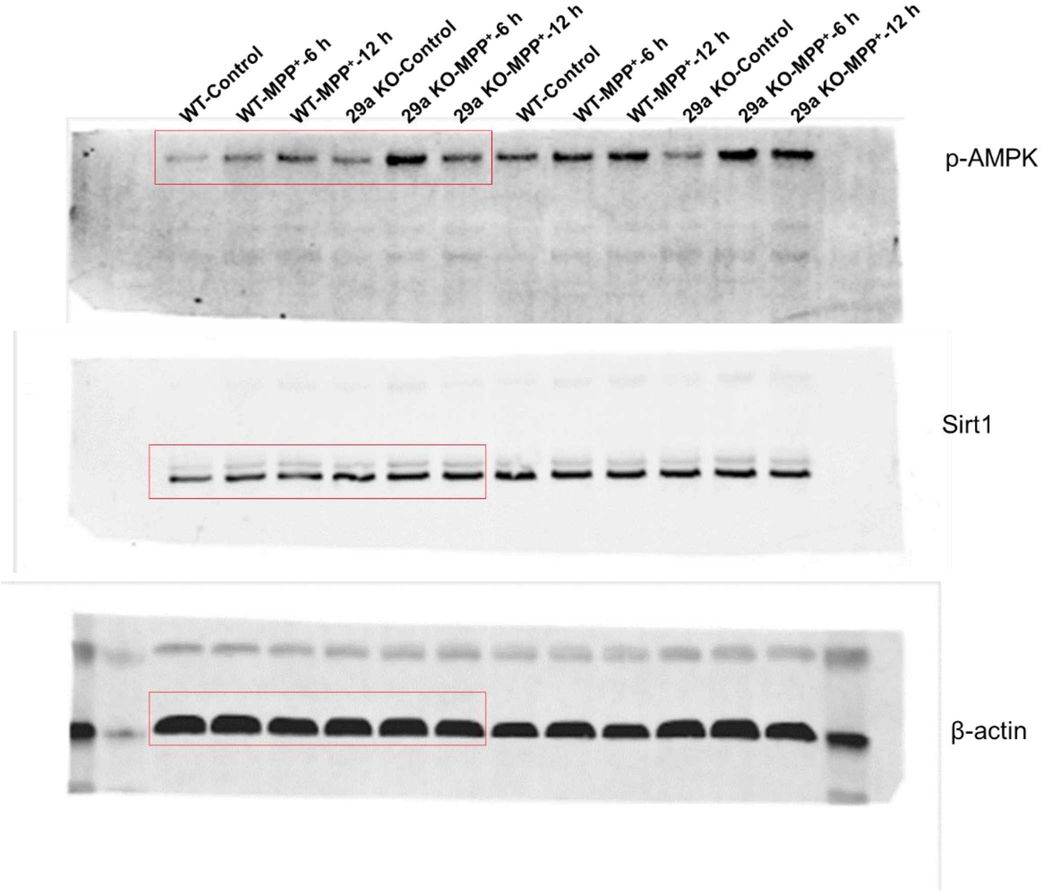

3. **Original images for Figure 6G**

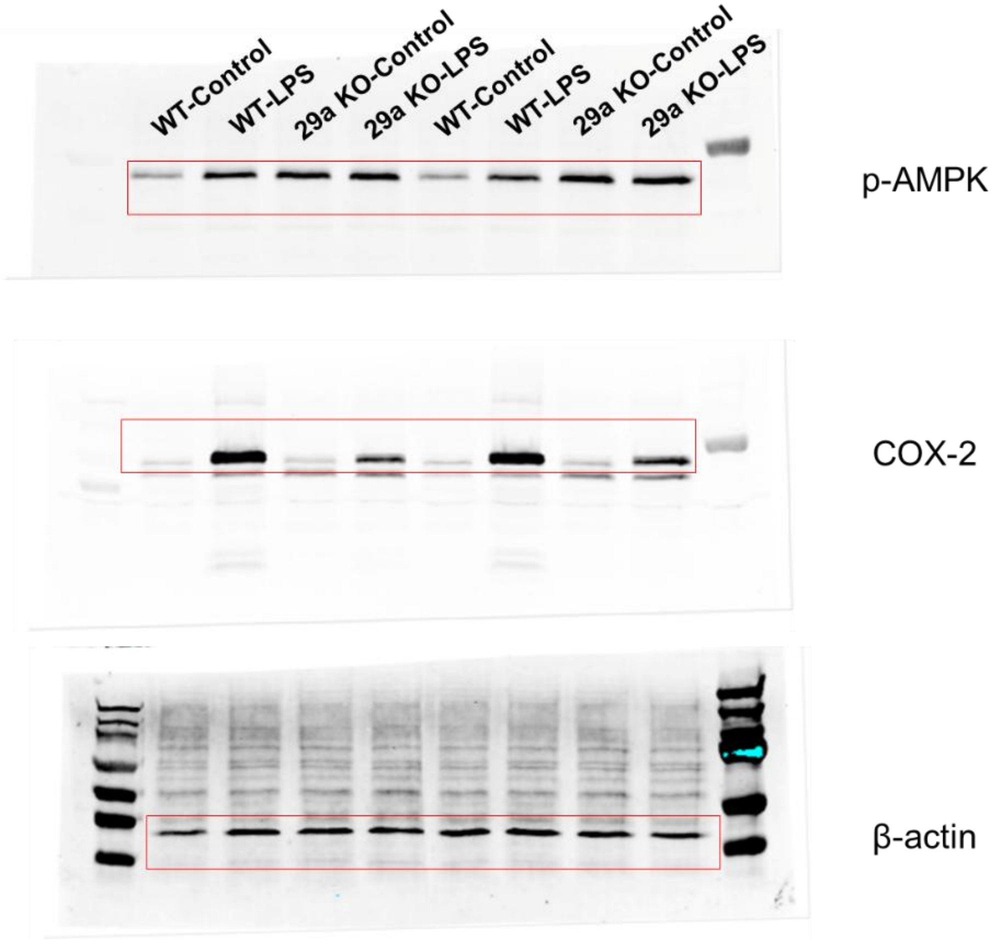

4. Original images for Figure 6H

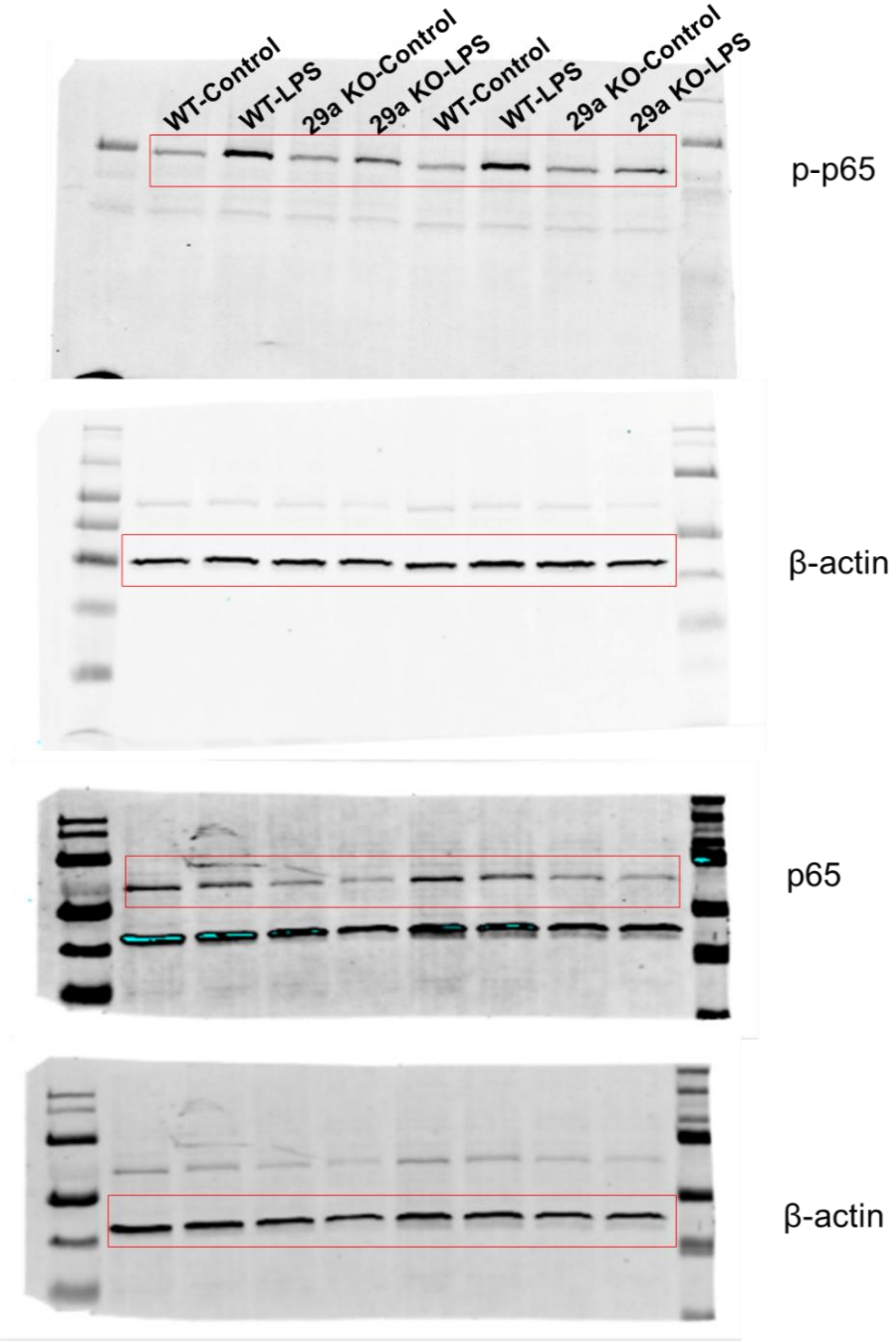

5. **Original image for Figure 7D**

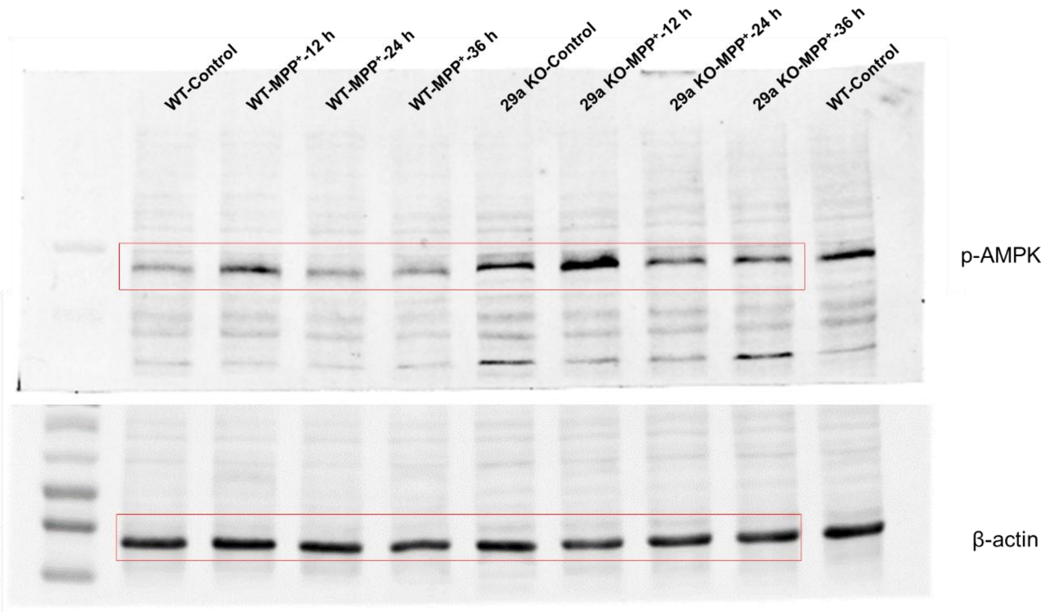

6. Original images for Figure S2B

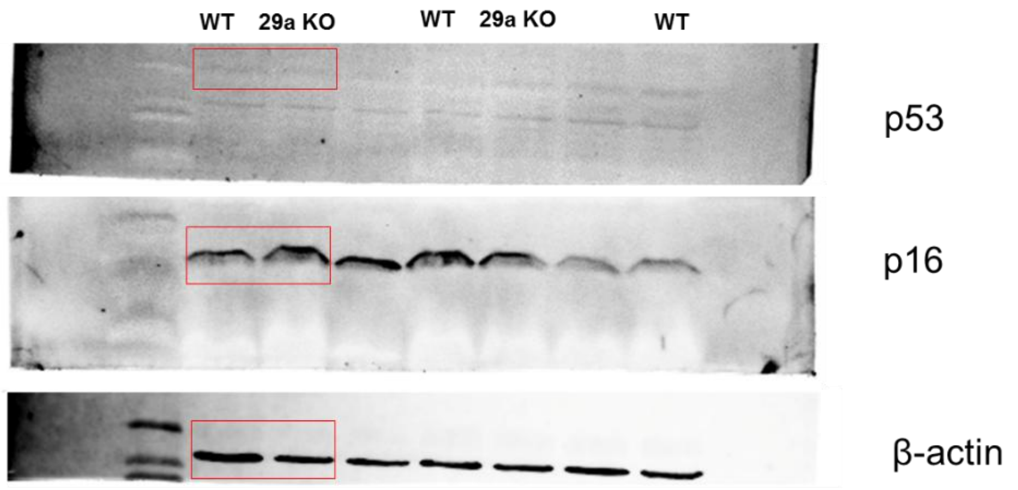

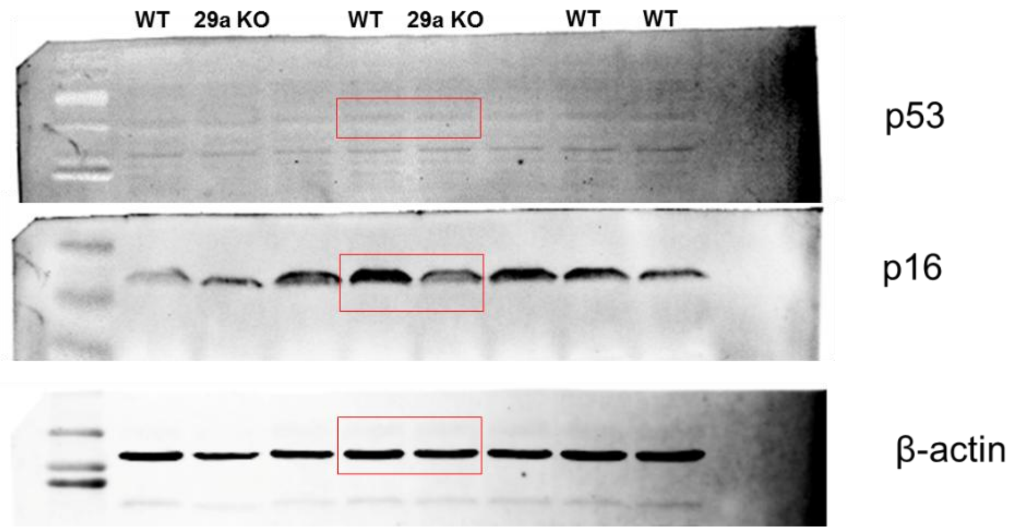

7. **Original image for Figure S8**

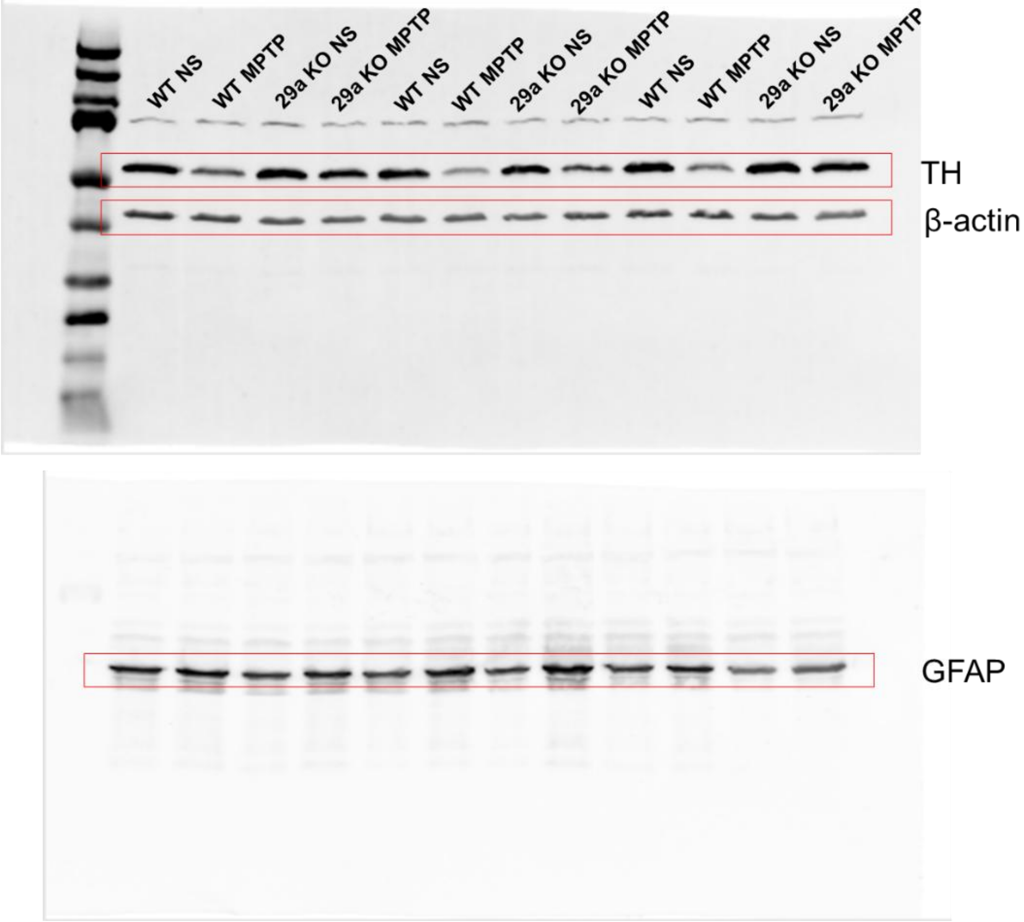

8. **Original image for Figure S11B**

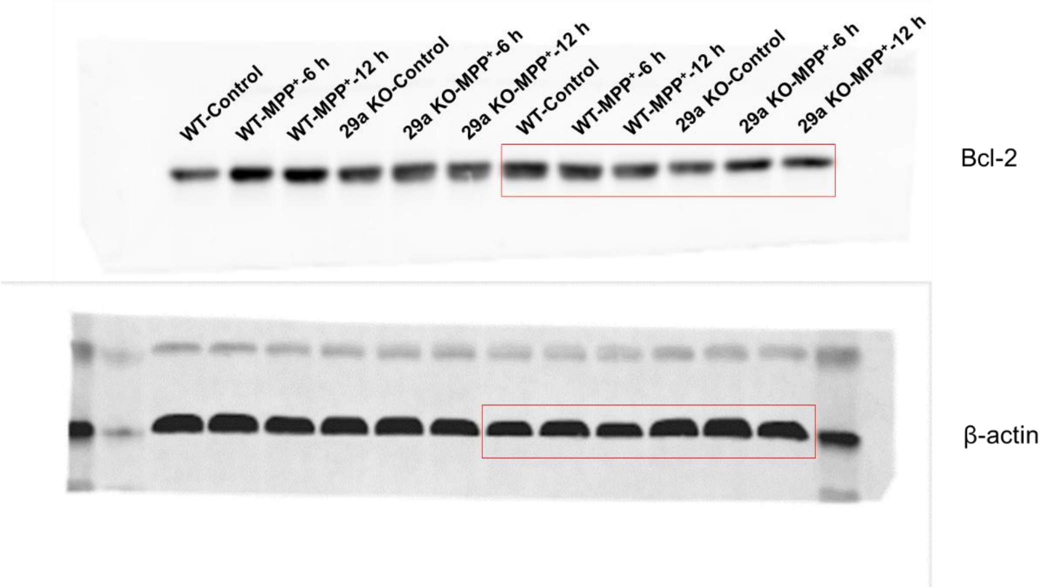

Supplement: Supplementary file 1 [file Data_Sheet_1.pdf]
